# Spatial Patterns of Diversity in Forest Birds of Peninsular India

**DOI:** 10.1002/ece3.72698

**Published:** 2025-12-16

**Authors:** Naman Goyal, Archita Sharma, Vishwa Jagati, Akshay Herur, Arunima Jain, Abhishek Gopal, Jahnavi Joshi, V. V. Robin

**Affiliations:** ^1^ Indian Institute of Science Education and Research Tirupati India; ^2^ CSIR‐Centre for Cellular and Molecular Biology Hyderabad India; ^3^ Academy of Scientific and Innovative Research (AcSIR) Ghaziabad India

**Keywords:** biogeography, birds, endemism, peninsular India, phylogenetic diversity

## Abstract

Biodiversity is structured via complex interactions among ecological, geological, and climatic processes. Regions with high heterogeneity in climate and topography are known to harbor fine‐scale patterns in diversity, which are often overlooked in global‐scale analyses. Here, we investigated the spatial phylogenetic patterns of forest bird diversity across peninsular India, a region characterized by high topographic and climatic heterogeneity. Using a comprehensive global bird phylogeny, we employed metrics such as Phylogenetic Diversity, Phylogenetic Endemism, Relative Phylogenetic Diversity and Relative Phylogenetic Endemism to quantify the evolutionary history and endemism of forest birds in peninsular India. We examined the roles of contemporary climate, historic climatic stability, and topography in shaping these patterns. Our results revealed a strong gradient in diversity, with the southern Western Ghats acting as a major hotspot for both taxonomic and phylogenetic diversity and endemism. We detected distinct ecological and phylogenetic community structures across the peninsula, likely shaped by regional species pools and biogeographic barriers. Areas with greater topographic complexity, higher precipitation, and greater historic climatic stability were found to support high diversity. Our study provides critical insights into biogeography in this understudied yet highly biodiverse region for forest birds.

## Graphical abstract

In this study, we analyzed patterns of diversity and endemism of forest birds across peninsular India, highlighting the influence of climate and topography. Our study shows that the southern Western Ghats are a major evolutionary hotspot for forest birds in peninsular India, and topographically complex and climatically stable areas support the highest forest bird diversity. These findings underscore the importance of regional environmental factors in driving biogeographic patterns in this understudied landscape.

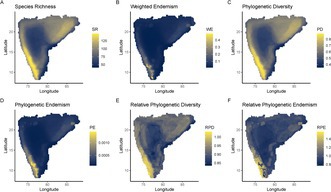

## 1 Introduction

Biodiversity is unevenly distributed across the globe, and there has been much interest in understanding the processes that drive these patterns. With unprecedented declines in biodiversity (Pereira et al. 2010), we are not only losing species, but also areas with distinct evolutionary history (Veron et al. 2017) and ecosystem services (Dobson et al. 2006). Hence, understanding the drivers of different facets of diversity is crucial for planning better conservation strategies.

Geological, ecological, and environmental processes are known to interactively structure patterns in diversity across space and time (Wiens and Donoghue 2004). Some of the key hypotheses explaining biodiversity patterns focus on topographic complexity, energy availability, and historical climatic stability. Mountains, for example, harbor higher‐than‐expected biodiversity due to steep climatic gradients over short distances and their role as barriers to dispersal, which promote isolation and species diversification (Rahbek, Borregaard, Antonelli, et al. 2019). Similarly, regions with high energy availability are expected to support greater diversity by providing the resources necessary for species coexistence (Araújo et al. 2008; Evans et al. 2005; Hawkins et al. 2003). Historical climatic stability is also a strong predictor of diversity, as reduced climatic fluctuations over evolutionary timescales allow for lineage persistence and accumulation (Araújo et al. 2008; Carnaval et al. 2009; Gómez et al. 2007; Wiens and Donoghue 2004). While these broad‐scale mechanisms are well recognized, understanding how they interact to influence regional patterns of biodiversity, particularly in topographically and climatically complex landmasses, remains a critical challenge.

Traditional methods of quantifying biodiversity and identifying conservation hotspots have largely relied on species richness (Myers et al. 2000). However, more recent approaches increasingly incorporate phylogenetic information to assess biodiversity patterns in an evolutionary context (Rosauer et al. 2009; Nitta et al. 2023; Paúl et al. 2023). These measures enable the assessment of evolutionary uniqueness in communities and the identification of regions with high evolutionary significance. Furthermore, they elucidate the influence of historical processes by revealing patterns in lineage diversification, extinction, and persistence (Rosauer et al. 2009; Nitta et al. 2023; Paúl et al. 2023). Phylogenetic Diversity and Phylogenetic Endemism are among the most widely used metrics for evaluating biodiversity through a phylogenetic lens. Phylogenetic Diversity quantifies the total branch length of a phylogeny connecting the species in an area (Faith 1992), while Phylogenetic Endemism weights those branch lengths by the relative geographic ranges of the species involved (Rosauer et al. 2009). Both metrics incorporate evolutionary history and provide valuable insights into how long‐term ecological and geological processes have shaped current biodiversity patterns.

**FIGURE 1:**
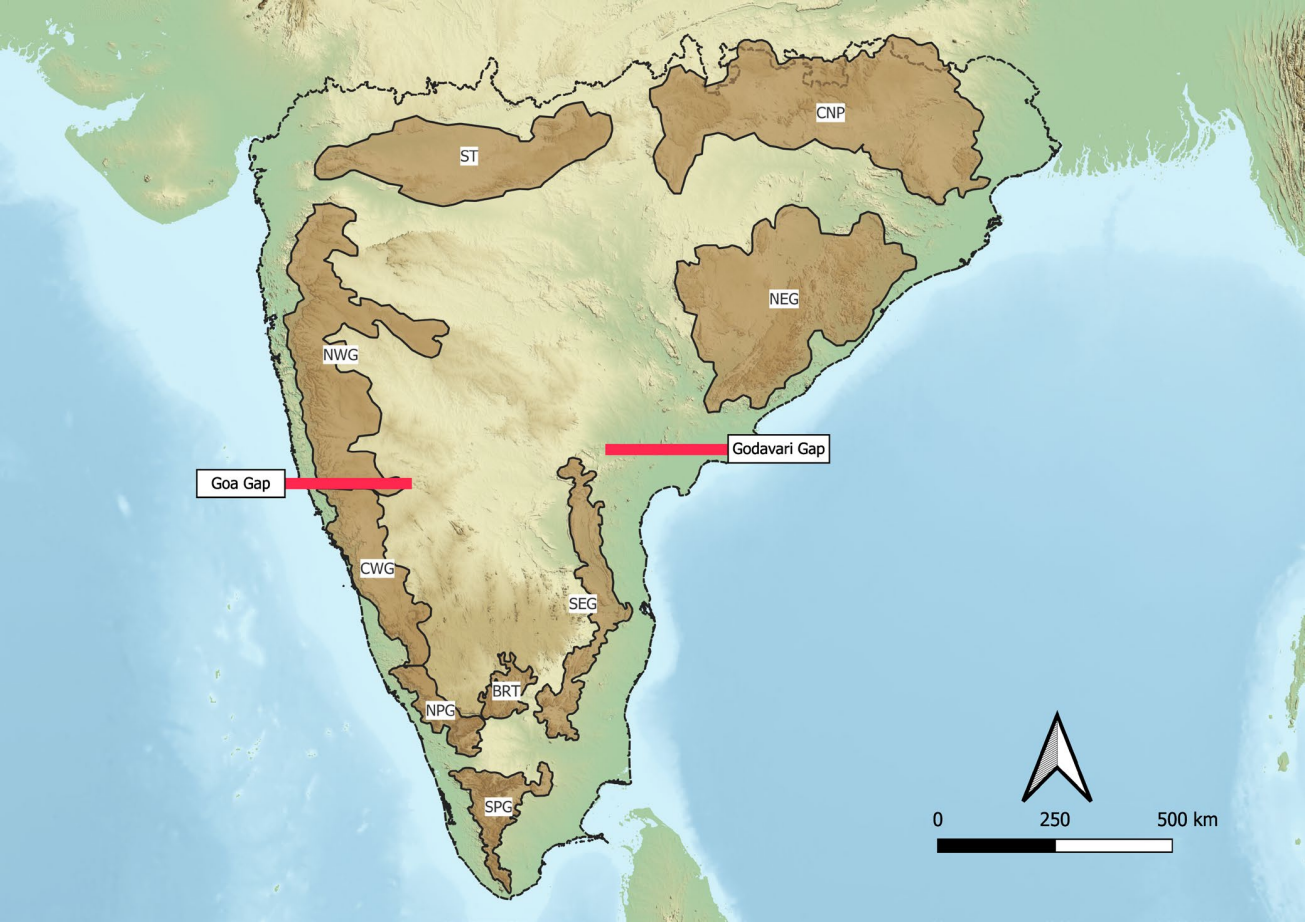
Map of peninsular India depicting major topographic zones (> 800 m elevation) ‐ the Western Ghats, divided into northern (NWG), central (CWG), and southern regions (NPG and SPG), the Eastern Ghats divided into north (NEG) and south (SEG), the Satpuras (ST), the Chota Nagpur Plateau (CNP), and the Biligirirangan Hills (BRT). The two major biogeographic divides, the Goa Gap and the Godavari Gap, are represented by the red lines.

Peninsular India is a region with a complex geoclimatic history and has high heterogeneity in topography and climate (Karanth 2017; Joshi and Karanth 2011). It consists of major topographical features such as the Deccan Plateau, the Western Ghats mountains, the Eastern Ghats mountains, and the Chota Nagpur Plateau (Figure 1). Furthermore, based on the Köppen climate classification, peninsular India consists of climatic zones ranging from tropical wet to arid climatic conditions (Beck et al. 2023; Jain et al. 2022).

Patterns in the distribution of biodiversity have been well documented in the Western Ghats, a global biodiversity hotspot in peninsular India. Studies on diversity patterns of several taxa show the presence of a latitudinal diversity gradient in the Western Ghats (Gopal et al. 2023; Bharti et al. 2021; Bose et al. 2019; Divya and Karanth 2021; Page and Shanker 2020; Davidar et al. 2005; Daniels 1992). Moreover, patterns in endemism for plants (Bose et al. 2019; Gopal et al. 2023), snails (Aravind et al. 2005), centipedes (Bharti et al. 2021), and amphibians (Daniels 1992) show that southern Western Ghats has a higher proportion of endemic species compared to its northern stretches. Further, the presence of old lineages of centipedes (Bharti et al. 2021) and both old and young lineages of plants (Gopal et al. 2023) supports the idea that southern Western Ghats has been a historic climatic refuge, acting both as a museum and cradle of diversity (Joshi and Karanth 2013). Environmental variation, topographic complexity, and historic climatic stability have played a role in driving these patterns in the WG (Page and Shanker 2020; Gopal et al. 2023; Bharti et al. 2021; Divya and Karanth 2021; Bose et al. 2019).

Our understanding of these patterns is limited at a broad peninsular India scale, with the inclusion of other mountain ranges such as the Eastern Ghats. Ramachandran et al. (2017) examined the distribution of birds at a subspecies level in peninsular India and found that several distribution boundaries coincided with physical or climatic divides within the peninsula, suggesting a role of both climate and topography in driving subspecies level distributional patterns. Two major divides identified in this study coincide with the Goa Gap (a strong climatic divide) and the Godavari Gap (both a climatic and physical divide), which had the highest turnover in the bird community in peninsular India. In addition, they find that wet regions that have been historically stable—potential refugia—harbor a higher proportion of endemism for birds. However, they relied only on subspecies level natural history information and did not incorporate phylogenetic information in assessing spatial patterns in diversity patterns for birds in peninsular India.

In this study, we investigated spatial phylogenetic patterns in the distribution of forest birds across peninsular India using the latest global bird phylogeny (McTavish et al. 2025). Specifically, we tested the roles of climate and topography in shaping these patterns. To do so, we employed a suite of phylogenetic metrics, Phylogenetic Diversity, Phylogenetic Endemism, Relative Phylogenetic Diversity, and Relative Phylogenetic Endemism, which allowed us to assess different aspects of evolutionary history and endemism within communities. While Phylogenetic Diversity and Phylogenetic Endemism help quantify the total evolutionary history and the geographic restriction of that history in a given region, Relative Phylogenetic Diversity and Endemism compare observed patterns to a null expectation (Mishler et al. 2014), thus highlighting regions with disproportionately old or young lineages. These metrics together enabled us to identify areas acting as evolutionary hotspots, detect potential refugia, and understand how historical processes have contributed to current biodiversity patterns in peninsular India, including less‐studied regions like the Eastern Ghats.

In particular, we focused on the following set of questions with specific expectations:

1. Do lower latitudes show greater diversity compared to the rest of peninsular India?

Based on the latitudinal diversity gradient hypothesis, we expected higher diversity in regions in the lower latitudes, such as the southern Western Ghats, compared to other regions in peninsular India.

2 Do different regions within the peninsula show differences in their community structure in an ecological and phylogenetic perspective?

Given that local community assemblages could be shaped by regional species pools (Cornell and Harrison 2014), we expected to find clusters of taxonomically and phylogenetically similar communities across the landscape. Identifying these patterns will provide insights into biogeographic regionalization within peninsular India using both taxonomic and phylogenetic information for species.

3 Do historic climatic stability, contemporary climate, and topography predict diversity across peninsular India?

Historic climatic stability, contemporary climate, and topography are known to drive patterns in diversity both at global scales and regional scales (Araújo et al. 2008; Gómez et al. 2007; Carnaval et al. 2009; Evans et al. 2005; Rahbek, Borregaard, Antonelli, et al. 2019). We examined the roles of historic climate stability, contemporary climate, and topography in driving the patterns in diversity for forest birds in peninsular India. We expected that areas with high topographical complexity, high precipitation (wetter areas), and high historical climatic stability would have higher diversity of forest birds.

## 2 Methods

### 2.1 Study Region and Species Selection

We define peninsular India as the inverted triangle part of the Indian subcontinent, bound by the Western Ghats in the west, the Eastern Ghats in the east, and the Satpuras and Chota Nagpur plateau in the north (Figure 1). There are approximately 600 species of birds known to occur in this region, with a slight variation in the number depending on the source of taxonomy. We further restricted our analyses only to the resident, breeding, terrestrial, forest bird species.

### 2.2 Species Distribution and Phylogenetic Data

We used the distribution maps for birds of the world from Birdlife International (BirdLife International, Handbook of the Birds of the World 2023) clipped to the extent of our study area (QGIS v 3.24.3; (QGIS Development Team 2024)). Furthermore, we filtered the dataset to include only birds that are native resident breeding species (values 1 and 2 under Birdlife metadata “Season”, and values 1 under “Origin”), and omitted migratory species that breed elsewhere from downstream analyses, resulting in 329 species. We used the AVONET dataset (Tobias et al. 2022) to classify the species based on the predominant habitat they use. Since the focus of this study was only on forest and woodland birds, we filtered the dataset to retain only species that predominantly use these habitats, including riverine habitats. Including species from non‐forest habitats would dilute the ecological signal and reduce the interpretability of the results. Filtering by forest and woodland habitat resulted in a total of 188 species (Table S1). We further renamed species following Clements/eBird 2023 taxonomy for downstream analyses to be able to use the latest bird phylogeny (McTavish et al. 2025). We pruned the phylogenetic tree to retain only the 188 species for downstream analyses (Figure S1). We also extracted the occurrence records for the final set of 188 species from the eBird dataset using custom scripts in R. This was done to compare how two different data sources for distribution influence the patterns of diversity.

### 2.3 Estimating Avian Diversity Metrics

To estimate the diversity metrics, we first generated presence‐absence matrices at 3 scales—10, 25, and 50 km. We chose these three relatively coarse scales as BirdLife range maps are based on expert opinion and may not represent fine‐scale species distributions (Warudkar et al. 2022). The presence‐absence matrices were generated (*lets.presab.grids* in the package LetsR (Vilela and Villalobos 2015)), and we estimated the Species Richness as the sum of all species present in each grid cell. Weighted Endemism was estimated by first calculating the inverse of the total number of grid cells occupied by each species across the study region, and then summing these values for all species present in each grid cell, as represented by the following equation:

$$\text{WE}=\sum_{i=1}^{s}\frac{1}{r_{i}}$$

where WE is the Weighted Endemism for a grid cell, *s* is the total number of species occurring in the grid cell, and *r* is the total number of the grid cells occupied by a species *i* in the study area. We estimated Phylogenetic Diversity, Phylogenetic Endemism, Relative Phylogenetic Diversity, and Relative Phylogenetic Endemism (*rand_test_cpr* of package *canaper* (Nitta et al. 2023)) simultaneously with testing for null models. For generating the null models, we initially used the “*swap*” algorithm from the package *vegan* (Oksanen et al. 2025). Briefly, this algorithm is a fixed‐fixed algorithm that swaps 2 × 2 submatrices to randomize the community matrix while maintaining the row and column totals (Gotelli and Entsminger 2003). To ensure that we have randomized our null models sufficiently, we ran optimization for *n_itr* and *n_reps* as per the recommendations (Nitta et al. 2023) (Figure S2). Based on our optimization results, we found that 200,000 *n_reps* and 1000 *n_itrs* were enough to randomize a community at a 25 × 25 km scale (Figures S2 and S3). Whereas *n_reps* had to be increased to 2,000,000 at a scale of 10 × 10 km to get a sufficiently randomized null community (Figure S3). We also assessed the nestedness in our data since fixed‐fixed algorithms struggle to randomize community matrices in cases where there is high nestedness in species communities (Ulrich and Gotteli 2007). Since we found high nestedness in our data (NODF = 88.66), we compared two additional randomization algorithms at a 25 km scale for generating null models, as implemented in the *vegan* package (Oksanen et al. 2025). (1) “*curveball*” which is another fixed‐fixed algorithm that selects two random rows in the matrix and finds a set of unique species in each row and redistributes them, preserving row and column totals (Strona et al. 2014). (2) “*r0*” which is a fixed‐equiprobable algorithm that maintains row totals but randomizes species in an equiprobable manner.

We also estimated Time‐Integrated Lineage Diversity using a custom R script. Time‐Integrated Lineage Diversity is a deep‐time complement to Phylogenetic Diversity, estimated by integrating the area under a lineage‐through‐time plot where the number of lineages is log‐transformed (Dexter et al. 2019). Further, to identify different types of endemism centers, we used a two‐step process called Categorical Analysis of Neo and Paleo Endemism (CANAPE) implemented in the package *canaper*. Briefly, this algorithm identifies grid cells with significantly high values (one‐tailed test, *α* = 0.05) in the numerator, denominator, or both components of the Relative Phylogenetic Endemism, indicating elevated endemism. Among these, grid cells are then classified based on the Relative Phylogenetic Endemism ratio (two‐tailed test, *α* = 0.05) into three categories: (1) paleo endemism (high Relative Phylogenetic Endemism) – an area with a high concentration of long range‐restricted branches, (2) neo endemism (low Relative Phylogenetic Endemism) – an area with a high concentration of short range‐restricted branches, and (3) mixed endemism (high in both numerator and denominator, but non‐significant Relative Phylogenetic Endemism ratio). Mixed endemism indicates a combination of rare long and short branches. Grid cells with high significance (*α* = 0.01) in both numerator and denominator were further designated as super endemism grid cells (Mishler et al. 2014; Nitta et al. 2023).

### 2.4 Assessing Turnover in Forest Birds Across Peninsular India

To assess the structure of communities across peninsular India for the selected species, we used a Grade of Membership model to cluster communities based on presence‐absence data, as implemented in the R package *Ecostructure* (White et al. 2019, 2021). Grade of Membership models allow units of analysis, such as geographic locations or species, to belong to multiple clusters (White et al. 2019, 2021). We ran this model for values of *K* from 2 to 9 with 10,000 iterations for each *K* and selected the best model based on estimated log likelihood for each *K*. We plotted the membership proportions using pie charts for each grid cell using the *ggplot2* package in R (Wickham 2016). Further, for each *K*, we identified the top species contributing to each cluster using the function *ExtractTopFeatures* in the *ecostructure* package (White et al. 2019, 2021) (Table S2). To evaluate the optimal value of *K* for peninsular India, we plotted the log likelihood values for each *K* from 2 to 9 and identified the optimal value of *K* where the increase in log likelihood starts flattening, that is, the elbow of the plot. This method is used regularly to estimate the optimal value of *K* in structure‐like methods in population genomics (Evanno et al. 2005).

Further, to assess the turnover in phylogenetic diversity across peninsular India, we estimated the phylogenetic beta diversity using Sorenson's Phylogenetic Index (Leprieur et al. 2012). We used the function *phylosor* from the package *picante* in R to generate a distance matrix of pairwise similarity between grid cells (Kembel et al. 2010) and *phylo.beta.pair* to estimate Simpson's pairwise phylogenetic dissimilarity from the package *betapart* in R. Further, we carried out a *K*‐means clustering using the distance matrix for values of *K* from 1 to 10 to identify regions of similar phylogenetic diversity. We calculated the total within‐cluster sum of squares (WSS) to evaluate the appropriate value for *K*. The WSS represents the sum of the squared distances between each data point and its assigned cluster centroid. We then plotted the WSS values against the corresponding *K* values, creating an elbow plot. The “elbow” point in the plot, where the rate of decrease in WSS sharply diminishes, was identified as the optimal *K*.

### 2.5 Environmental Correlates of Avian Diversity

We used a set of climatic and topographic variables that capture the spatial and temporal heterogeneity in the region and are known to influence patterns in the distribution of birds (Acevedo and Sandel 2021; Ramachandran et al. 2017) (Table 1). The data were available at 1 km × 1 km resolution and aggregated to 25 km × 25 km resolution. We checked for autocorrelation for all the variables using the *corrplot* package in R and selected variables that had a Pearson's correlation value of less than 0.5 or more than −0.5. In total, we selected 12 climatic and topographic variables.

**TABLE 1:** List of climatic and topographic variables used in this study to identify the drivers of species richness, phylogenetic diversity, and phylogenetic endemism in forest birds of peninsular India.

Type	Variable	Description	Source
Climatic	Bio9	Mean Temperature of Driest Quarter	Karger et al. (2017)
	Bio10	Mean Temperature of Warmest Quarter	Karger et al. (2017)
	Bio11	Mean Temperature of Coldest Quarter	Karger et al. (2017)
	Bio16	Precipitation of Wettest Quarter	Karger et al. (2017)
	Bio17	Precipitation of Driest Quarter	Karger et al. (2017)
	Bio18	Precipitation of Warmest Quarter	Karger et al. (2017)
	TCC mean	Mean of Total Cloud Cover	Wilson and Jetz (2016)
	TCC sd	Standard Deviation of Total Cloud Cover	Wilson and Jetz (2016)
	LGM_CCV	Climate Change Velocity since Last Glacial Maxima (LGM)	This study
Topographic	Aspect cosine	Cosine of Aspect	Amatulli et al. (2018)
	Aspect sine	Sine of Aspect	Amatulli et al. (2018)
	Elevation	Elevation relative to mean sea level	Amatulli et al. (2018)
	TRI	Topographic Ruggedness Index	Amatulli et al. (2018)

To estimate the climatic stability of the region since the last glacial maximum (LGM), we relied on two main variables—the Annual Mean Temperature (Bio1) and Annual Precipitation (Bio12). We obtained the data at 1 km × 1 km resolution at 100‐year time intervals from the Trace21k dataset (Karger et al. 2023). We used climate change velocity to measure the climatic stability of the study area since the LGM. Climate change velocity is a measure of local climatic displacement over an area and is calculated by dividing the change in climate over time by the change in climate over space for a given area (Loarie et al. 2009). Climate change velocity is a measure of the rate at which species would have to shift their ranges to track changes in climate (Loarie et al. 2009; Sandel et al. 2011). The data was first aggregated to a 25 km × 25 km scale in R using the package *raster*. We then used the package *VoCC* in R to estimate the gradient‐based climate change velocity (García Molinos et al. 2019). We first estimated the climate change velocity for Bio1 and Bio12 separately, then rescaled the velocities to a scale of 0 to 1 by normalizing the resultant rasters. Here, 0 means the lowest velocity or a climatically stable region, and 1 means the highest velocity of climate change or a climatically unstable region. We finally combined both temperature and precipitation climate change velocities into a single raster by adding the layers together. We checked for correlation between climate change velocity and the 12 selected variables using the same method detailed above.

We assessed the role of climatic and topographic variables at a 25 × 25 km scale. First, we scaled all the predictor variables and ran linear regression models for Species Richness, Phylogenetic Diversity, and Phylogenetic Endemism. Second, we ran 3 sets of models for each response variable: (1) with only climatic variables, (2) with only topographic variables, and (3) combined climatic and topographic variables. We tested for spatial independence in residuals for all the linear regression models using the function *correlog* of the package *pgirmess* in R to estimate Moran's I statistic at different distance classes (Giraudoux 2013). Since we found significant spatial autocorrelation in the residuals (*p* < 0.05), we used spatial autoregressive models (Kissling and Carl 2008). For each model, we identified all neighbors up to the first non‐significant value in our correlog plots using the function *dnearneigh* in the *spdep* package in R (Bivand and Piras 2015). We then generated a spatial weights matrix using the function nb2listw using a standard coding scheme (Kissling and Carl 2008). We tested multiple spatial models in a model selection framework implemented in the *spdep* package in R. The models were then ranked according to their model performance using Akaike Information Criterion (AIC) scores (Table S3).

## 3 Results

### 3.1 Patterns in Species Richness, Phylogenetic Diversity, and Phylogenetic Endemism

**FIGURE 2:**
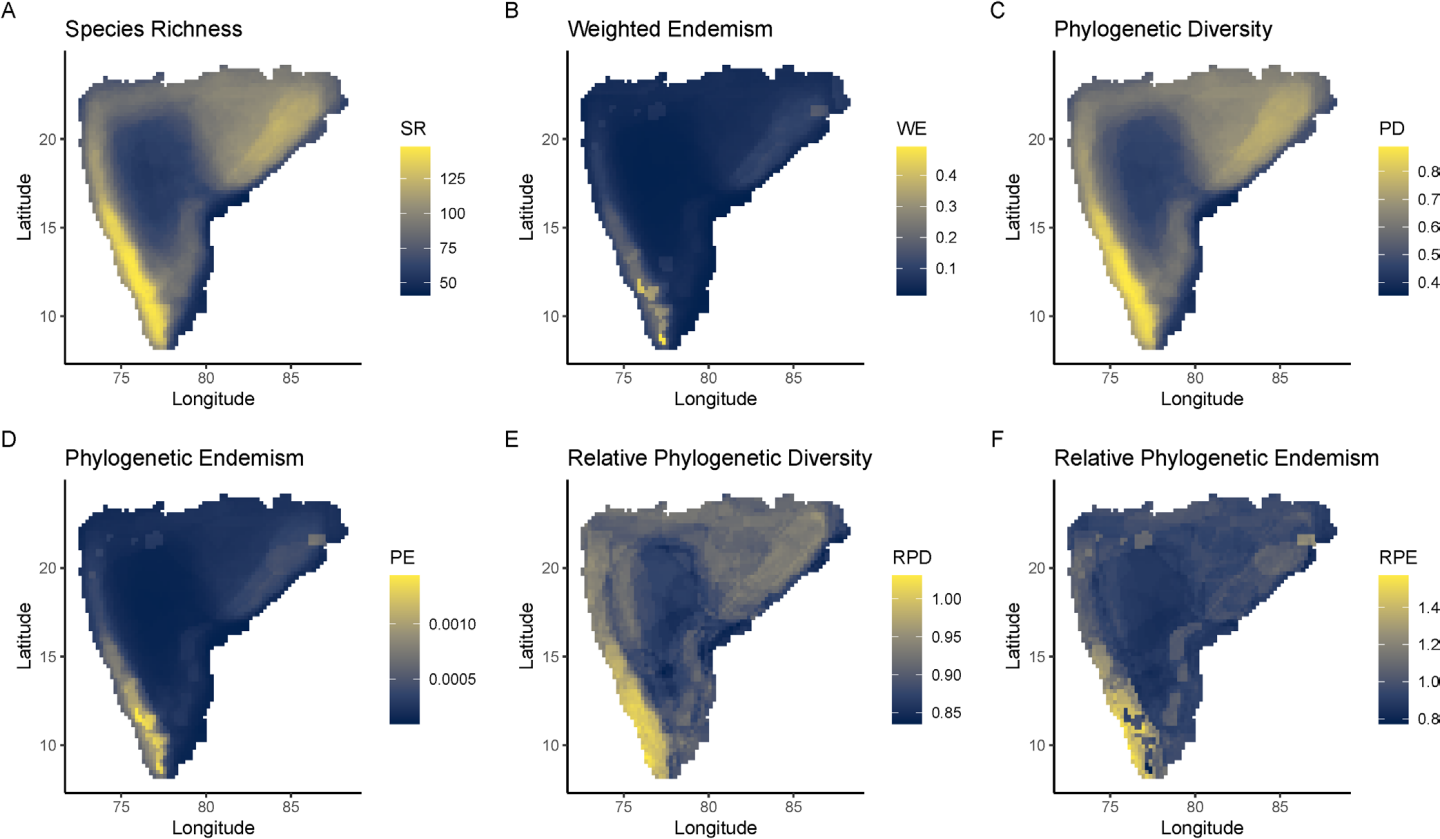
Spatial patterns of forest bird diversity across peninsular India at a 25 km × 25 km resolution based on BirdLife International range maps. (A) Species Richness (SR), (B) Weighted Endemism (WE), (C) Phylogenetic Diversity (PD), (D) Phylogenetic Endemism (PE), (E) Relative Phylogenetic Diversity (RPD), and (F) Relative Phylogenetic Endemism (RPE).

Our results indicate that the highest Species Richness and Weighted Endemism were found to be within the southern Western Ghats (Figure 2A,B). Overall, we observed a pattern consistent with a latitudinal diversity gradient, where lower latitudes have higher species richness of forest birds than the higher latitudes in the Western Ghats. However, in the case of the Eastern Ghats, the pattern is reversed, where higher species richness is observed in high latitudes (Figure 2A).

Patterns in Phylogenetic Diversity also showed similar patterns to Species Richness (Figure 2C). The Western Ghats showed a latitudinal gradient in Phylogenetic Diversity with higher values at lower latitudes. The hill ranges like Satpuras, Chota Nagpur Plateau, and the Eastern Ghats also showed relatively high Phylogenetic Diversity compared to parts of the rest of peninsular India. Linear regression examining relationships between Species Richness and Phylogenetic Diversity showed that they are significantly positively correlated (Figure S4). Patterns in Phylogenetic Endemism showed a latitudinal gradient in Western Ghats, with the southern Western Ghats having the highest Phylogenetic Endemism in peninsular India (Figure 2D).

We found the highest Relative Phylogenetic Diversity and Endemism values in the southern Western Ghats, but other areas like the northern Western Ghats, the Eastern Ghats, the Chota Nagpur Plateau, and parts of the Satpura Hills also showed relatively high Relative Phylogenetic Diversity and Endemism (Figure 2E,F). The results indicate that areas such as the southern Western Ghats, having both high Relative Phylogenetic Diversity and Endemism, have a higher accumulation of range‐restricted ancient lineages of forest birds. Moreover, we did not find a strong effect of the choice of randomization algorithm for the null model on patterns in Relative Phylogenetic Diversity and Endemism (Figure S15).

The endemism type classification showed a strong impact of the null model algorithm choice (Figure S16). Across all comparisons, we find that several grid cells within the southern Western Ghats were identified to be centers of neo, paleo, super, and mixed endemism (Figures S9 and S16), indicating a significantly high proportion of both range‐restricted ancient and young lineages of forest birds. There were some differences in the patterns of endemism type depending upon the choice of null model for other parts of peninsular India (Figure S16). We found that the northern Eastern Ghats and the Satpuras have grid cells exhibiting mixed endemism. The endemism type classification using the “*r0*” algorithm identified several more grid cells across the Western Ghats and the northern Eastern Ghats as centers of neo, super, paleo, and mixed endemism types compared to the fixed‐fixed algorithms (Figure S16C). The significance of Relative Phylogenetic Diversity showed higher than expected values in the southern Western Ghats for all three null models (Figure S17A–C). The significance of Relative Phylogenetic Endemism also displayed higher than expected values in the southern Western Ghats, though the “*r0*” algorithm identified several more grid cells with lower than expected values across peninsular India (Figure S17D–F). These results highlight that the southern Western Ghats have a significant accumulation of ancient range‐restricted lineages for birds.

### 3.2 Patterns in Community Structure

**FIGURE 3:**
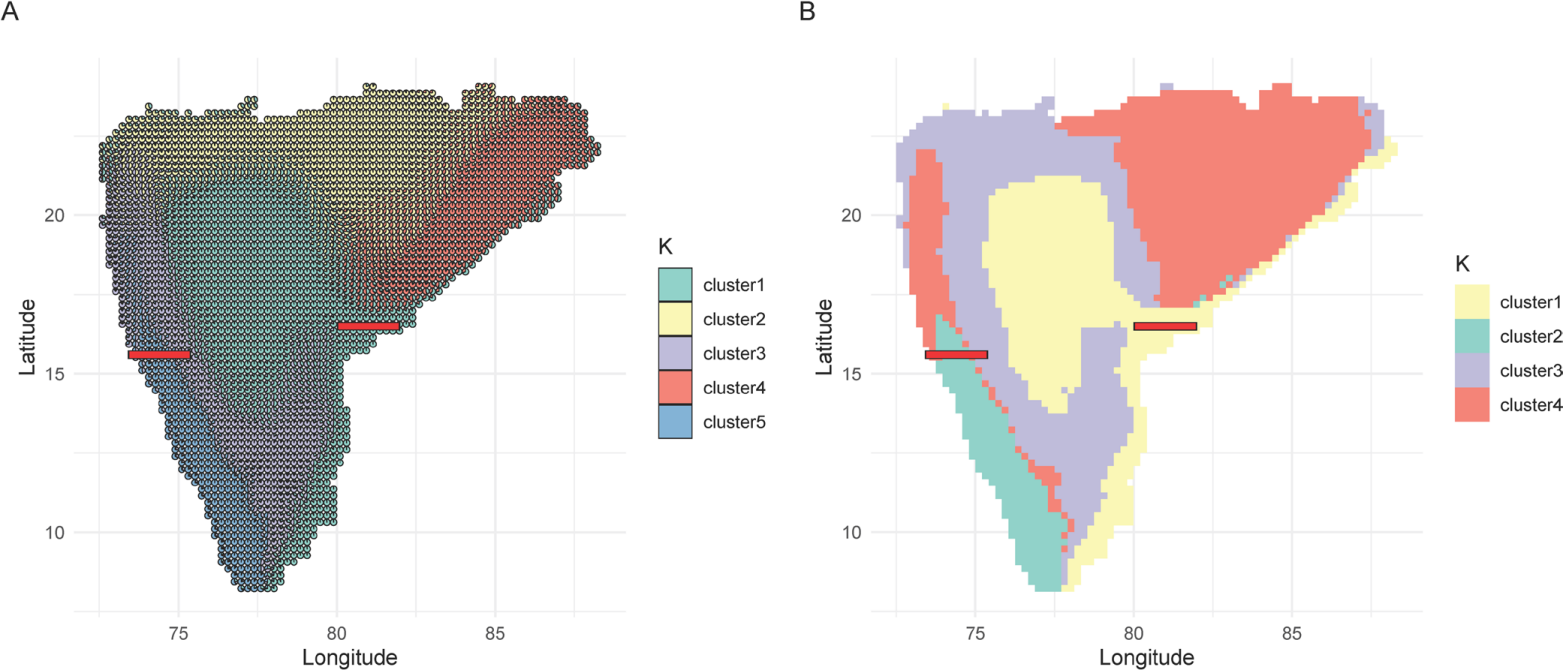
Spatial patterns of forest bird community structure across peninsular India. (A) Taxonomic community structure based on a Grade of Membership model (*K* = 5), where pie charts show proportions of each cluster at each grid cell. (B) Phylogenetic community structure derived from *K*‐means clustering of Sorenson's phylogenetic beta diversity (*K* = 4). Red lines represent known biogeographic barriers, Goa Gap and Godavari Gap (Ramachandran et al. 2017).

We observed distinct forest bird communities across peninsular India, both in terms of species composition and phylogenetic similarity (Figure 3). At lower values of *K*, regions such as the Western Ghats, Satpuras, Chota Nagpur Plateau, and Eastern Ghats segregated from the rest of Peninsular India, highlighting the distinctiveness of hill range communities (Figures S12–S14). With increasing *K*, finer scale partitioning emerged within these ranges. At the optimal clustering value of *K* = 5 for species turnover (Figure S10) and *K* = 4 for phylogenetic turnover (Figure S11), the Deccan Peninsula and the eastern coastal plains formed a single community, while the hill ranges (Western Ghats, Eastern Ghats, Satpuras, and Chota Nagpur Plateau) each formed distinct clusters (Figure 3A,B). Additionally, we observed turnover within the Western Ghats across the Goa Gap and within the Eastern Ghats across the Godavari Gap. When analyzing only the turnover component of phylogenetic beta diversity, similar patterns emerged: the Western Ghats and other hill ranges segregated at low *K* values, while at *K* = 4, the Western Ghats formed a single phylogenetically coherent community, and the Satpuras and Chota Nagpur Plateau clustered with the northern Eastern Ghats (Figure S14). Turnover across the Goa Gap within the Western Ghats becomes evident only at higher clustering levels (*K* = 8; Figure S14).

### 3.3 Predictors of Diversity Metrics

**FIGURE 4:**
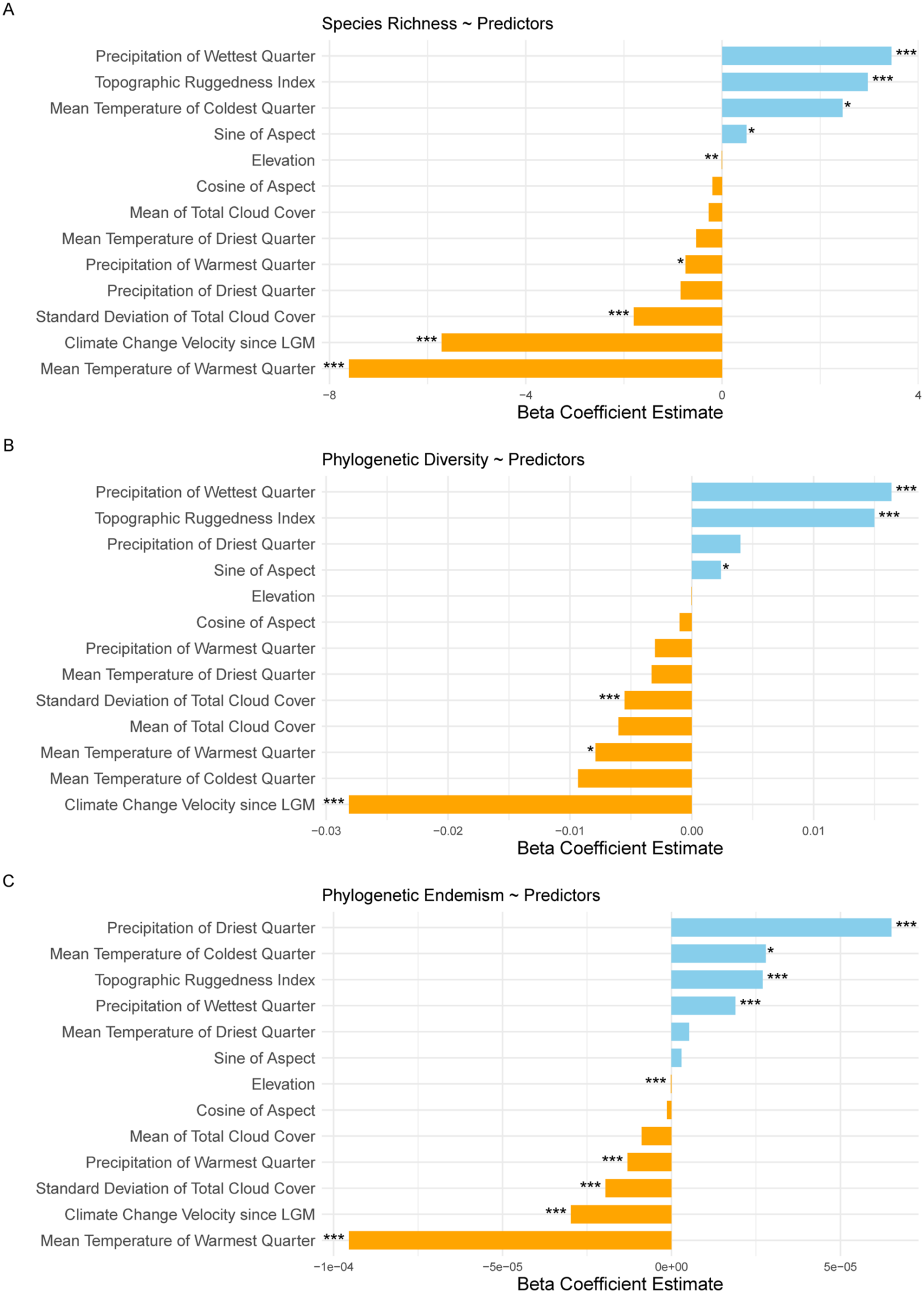
Estimates of coefficients for the best spatial autoregressive models for (A) Species Richness (SR), (B) Phylogenetic Diversity (PD), and (C) Phylogenetic Endemism (PE), incorporating climate and topographic variables. Blue bars indicate positive correlations, and orange bars indicate negative correlations. Significance: ***p < 0.001, **p < 0.01, *p < 0.05.

The best spatial autoregressive models for Species Richness, Phylogenetic Diversity, and Phylogenetic Endemism included both climate and topographic variables based on AIC scores. Across all three metrics, Precipitation of Wettest Quarter and Topographic Ruggedness Index were consistent positive correlates, while Climate Change Velocity since the Last Glacial Maximum and Mean Temperature of Warmest Quarter were significant negative correlates (Figure 4).

For Species Richness, additional positive correlates included the Sine of Aspect, whereas Mean of Total Cloud Cover and its seasonality (Standard Deviation) had negative effects (Figure 4A). The model for Phylogenetic Diversity showed a similar pattern, with Mean of Total Cloud Cover having a negative effect (Figure 4B). For Phylogenetic Endemism, Precipitation of Driest Quarter and Mean Temperature of Coldest Quarter were also significant positive correlates, whereas the Standard Deviation of Total Cloud Cover had negative effects on Phylogenetic Endemism (Figure 4C).

### 3.4 Results Across Scales and Datasets

The results at all three scales—50 km × 50 km, 25 km × 25 km, and 10 km × 10 km—were mostly concordant (Figure 2, Figures S5–S7). All the estimates for Species Richness, Weighted Endemism, Phylogenetic Diversity, Phylogenetic Endemism, Relative Phylogenetic Diversity, Relative Phylogenetic Endemism, and Time‐Integrated Lineage Diversity were consistent with the highest diversity in the southern Western Ghats compared to the rest of the peninsula. Moreover, with eBird data at a 25 km × 25 km scale, the patterns in these metrics were similar to results with birdlife range maps; however, there are several gaps for our species, which may yield underestimated ranges (Figure S8). Hence, we focused our discussion based on birdlife range maps at a scale of 25 km × 25 km.

## 4 Discussion

Our study quantified spatial patterns in Species Richness, Weighted Endemism, Phylogenetic Diversity, and Phylogenetic Endemism for forest birds of peninsular India. Overall, our results indicated that the diversity of forest birds is uneven across peninsular India, with regions like the southern Western Ghats being an evolutionary hotspot for forest birds. Moreover, we identified distinct biogeographic zones within the peninsula based on both species composition and phylogenetic similarity. These zones coincide with known biogeographic barriers, highlighting the role of such barriers in shaping the current patterns in bird diversity. We also found support for historic climatic stability and topography playing important roles in driving these patterns in diversity.

### 4.1 Spatial Patterns in the Diversity of Forest Birds

The southern Western Ghats had the highest Species Richness, Phylogenetic Diversity, Weighted Endemism, Phylogenetic Endemism, and Time‐Integrated Lineage Diversity across the entire peninsula. Several species of forest birds, such as Gray‐headed Bulbul *Microtarsus priocephalus*, Flame‐throated Bulbul *Rubigula gularis*, and two genera of birds, *Sholicola* and *Montecincla*, are endemic to the southern Western Ghats (Robin et al. 2017). These species are evolutionarily distinct and occur in small geographic areas, contributing to high Phylogenetic Endemism in the southern Western Ghats. Further, our results indicated a latitudinal diversity gradient within the Western Ghats, with the southern Western Ghats having higher diversity across all metrics, decreasing towards higher latitudes. This pattern is known for several taxa in the Western Ghats, such as centipedes (Bharti et al. 2021), plants (Gopal et al. 2023; Page and Shanker 2020; Divya and Karanth 2021; Davidar et al. 2005), amphibians (Daniels 1992), and snails (Aravind et al. 2005). Moreover, the southern Western Ghats also had high Relative Phylogenetic Diversity and Endemism for forest birds—indicative of the accumulation of several old and range‐restricted lineages. Similar patterns have been seen in the case of centipedes (Bharti et al. 2021) and plants (Gopal et al. 2023) in the Western Ghats, with high proportions of old lineages concentrated in the south. Overall, our results support the idea that the southern Western Ghats act as a climatic refuge even for birds, allowing the persistence of old lineages over evolutionary timescales.

However, the Eastern Ghats showed a reverse pattern, with northern regions having relatively higher Species Richness, Weighted Endemism, Phylogenetic Diversity, Phylogenetic Endemism, and Time‐Integrated Lineage Diversity than the southern Eastern Ghats. The northern Eastern Ghats and Chota Nagpur Plateau form interesting regions from a biogeographic perspective because of their proximity to the Himalayas. Several Himalayan elements, such as Crimson Sunbird *Aethopyga siparaja* , Lineated Barbet *Psilopogon lineatus* , Gray Treepie *Dendrocitta formosae* , and Abbott's Babbler *Malacocincla abbotti* , are known to have disjunct distributions within these regions. These elements do not extend further into any other part of the peninsula, contributing to the uniqueness of these regions. Furthermore, the northern Eastern Ghats and Chota Nagpur Plateau form a topographically complex region with relatively high rainfall compared to the southern Eastern Ghats due to receiving rainfall from both the southwest and northeast monsoons (Ramdas 1974).

Regions like the central Indian highlands (including the Satpuras and the Chota Nagpur Plateau) and the northern Eastern Ghats, although they showed relatively high Species Richness, Phylogenetic Diversity, and Time‐Integrated Lineage Diversity, the Weighted and Phylogenetic Endemism in these regions was very low compared to regions like the southern Western Ghats. The occurrence of very few endemic and range‐restricted lineages, compared to the southern Western Ghats, may drive these patterns in endemism across the peninsula. These patterns were consistent even for Relative Phylogenetic Diversity and Endemism in these regions. Although Relative Phylogenetic Diversity was relatively high in northern Western Ghats, central Indian highlands, and the northern Eastern Ghats, Relative Phylogenetic Endemism was low in these regions. This indicates that, though there is a high proportion of old lineages in these regions, most of them are widespread and not range‐restricted. These patterns align with findings from central Indian tropical dry forests, where phylogenetic community analyses of woody trees revealed greater evolutionary distinctiveness in low‐elevation plots (Grant et al. 2023).

Categorical analyses of neo and paleo endemism delineated centers of endemism across peninsular India for forest birds. Some sites in the Satpuras, Chota Nagpur Plateau, and the northern Eastern Ghats showed a mixed endemism type, indicating a mix of both young and old lineages of range‐restricted species in these regions. While most of peninsular India had non‐significant endemism, the southern Western Ghats emerged as a major center of endemism, making it an evolutionary hotspot for forest birds. Areas within the southern Western Ghats showed sites with a significant mix of both young and old range‐restricted lineages (neo, paleo, super, and mixed endemism types). These patterns are consistent with studies on other taxa, such as centipedes (Bharti et al. 2021; Joshi and Karanth 2013) and plants (Gopal et al. 2023), where both old and young range‐restricted lineages contribute to high diversity in the southern Western Ghats.

We acknowledge that the choice of the null model has a critical influence on the classification of endemism types. Fixed‐fixed algorithms, such as “*swap*” and “*curveball*”, preserve both site richness and species occurrence frequencies, which makes them more conservative and potentially underestimate grid cells with significant endemism (Ulrich and Gotteli 2007; Gotelli and Ulrich 2011; Molina and Stone 2020). Conversely, the “*r0*” algorithm, which preserves only site richness and allows species frequencies to vary freely, is relatively less restrictive and highlights more grid cells in the Western Ghats and the northern Eastern Ghats with neo, paleo, super, and mixed endemism types. Conservatively, these patterns emphasize that the southern Western Ghats are a major center of forest bird endemism, with additional, but more spatially scattered, endemism hotspots identified in other topographically complex, high diversity regions of peninsular India.

### 4.2 Community Structure and Composition Across Peninsular India

Our results for taxonomic and phylogenetic turnover in peninsular India identified multiple distinct clusters. We observed that the northern Eastern Ghats and central and southern Western Ghats formed distinct clusters, whereas the southern Eastern Ghats and the northern Western Ghats showed some similarities in their communities. Satpuras and the Chota Nagpur Plateau formed a distinct cluster as well. These patterns were consistent even in the phylogenetic turnover in communities. The turnover was greatest across the Goa Gap and the Godavari Gap in both taxonomic and phylogenetic perspectives. The Godavari Gap is a known biogeographic barrier for birds, with several sister species split across this barrier (Ramachandran et al. 2017; Ripley and Beehler 1990). Moreover, several Himalayan elements, such as Crimson Sunbird *Aethopyga siparaja* , Lineated Barbet *Psilopogon lineatus* , Gray Treepie *Dendrocitta formosae* , and Abbott's Babbler *Malacocincla abbotti* , have their southernmost distribution limited to the north of the Godavari Gap. Such species contribute to the uniqueness of the communities of forest birds in the northern Eastern Ghats and central Indian highlands. Similarly, several Western Ghats endemics, such as Dark‐fronted Babbler *Dumetia atriceps*, Wayanad Laughingthrush *Pterorhinus delesserti*, Gray‐headed Bulbul *Microtarsus priocephalus*, and Flame‐throated Bulbul *Rubigula gularis*, are known to have their distributional ranges restricted to the south of the Goa Gap (Ramachandran et al. 2017). Although a previous study on centipedes revealed turnover in communities across the Palghat Gap in southern Western Ghats (Bharti et al. 2021), we recovered no such pattern at any value of *K* for both taxonomic and phylogenetic turnover in our study. This may be surprising when we consider the fact that the Palghat Gap is a well‐known biogeographic barrier in the southern Western Ghats for several taxa (Joshi and Karanth 2013; Biswas and Karanth 2021), including birds (Robin et al. 2015). However, we believe that this can potentially be an effect of the coarseness of the study scale and expert‐drawn range maps for birds, and investigations at a finer scale may reveal community structure in forest birds across the Palghat Gap. Several potential biogeographic units have been identified for birds across peninsular India by investigating turnovers in birds at a smaller taxonomic unit of subspecies (Ramachandran et al. 2017). Our results are mostly concordant with these patterns, depicting the strongest taxonomic turnover across the Goa Gap and Godavari Gap in peninsular India. Even from a phylogenetic perspective, our results showed the effects of the Goa Gap and the Godavari Gap in shaping patterns in phylogenetic similarity in forest bird communities in peninsular India, reinforcing the importance of biogeographic barriers in shaping community structure. However, it is important to note that several species from peninsular India do not have genetic datasets available, and hence the diversity in the region may be underestimated due to the potential of cryptic species within the region (Reddy 2014).

### 4.3 Correlates of Forest Bird Diversity in Peninsular India

We tested the role of topography and climate in driving patterns in Species Richness, Phylogenetic Diversity, and Phylogenetic Endemism for forest birds in peninsular India. Our results indicated that both topography and climate play an important role in driving patterns in these diversity metrics.

The Topographic Ruggedness Index was found to be a strong and significant positive predictor of all three diversity metrics for forest birds. Regions with high topographic complexity in peninsular India have high Species Richness, Phylogenetic Diversity, and Endemism. These topographically complex regions coincide with major hills in peninsular India. Global‐scale studies have shown that mountains tend to harbor high diversity (Rahbek, Borregaard, Antonelli, et al. 2019; Rahbek, Borregaard, Colwell, et al. 2019) compared to nearby lowlands and less topographically complex areas. Mountains, with their complex topography, have high climatic heterogeneity over small spatial scales and act as barriers to dispersal, ultimately promoting the process of speciation (Rahbek, Borregaard, Antonelli, et al. 2019; Rahbek, Borregaard, Colwell, et al. 2019). The Western Ghats in peninsular India have high topographical complexity in lower latitudes compared to higher latitudes. Since the complexity of topography was found to be a significant positive predictor for Species Richness, Phylogenetic Diversity, and Endemism, the decrease in diversity in higher latitudes of Western Ghats may be the result of reduced topographical complexity in higher latitudes. A similar trend is seen even in the case of plants, where higher evolutionary diversity is seen in topographically heterogeneous stretches of the Western Ghats (Gopal et al. 2023). The role of topography in driving patterns in Species Richness and Phylogenetic Diversity can be extended even to the Eastern Ghats. Higher Species Richness and Phylogenetic Diversity in northern Eastern Ghats, as opposed to southern regions, as expected under predictions from the latitudinal diversity gradient, can be explained by heterogeneity in topography across the Eastern Ghats. The northern Eastern Ghats have a higher topographic ruggedness compared to the southern regions, which may potentially drive the pattern of a reversed latitudinal diversity gradient in the EG for forest birds. This, coupled with the geographic proximity of the northern Eastern Ghats to the Himalayas, may play a crucial role in the persistence of high diversity within the region.

Our results also highlighted a major role of climate in driving patterns in these diversity metrics. Regions with high rainfall (high Precipitation of the Wettest Quarter and Precipitation of Driest Quarter), moderately warmer climate (high Mean Temperature of Driest Quarter and Mean Temperature of Coldest Quarter, but low Mean Temperature of Warmest Quarter), and historically stable climate (low Climate Change Velocity since Last Glacial Maxima) tend to have higher Species Richness, Phylogenetic Diversity, and Endemism. Our results are in line with expectations that stable, warm, and wet climate—climatic refugia—supports higher diversity (Araújo et al. 2008; Carnaval et al. 2009; Gómez et al. 2007; Wiens and Donoghue 2004). Regions like the southern Western Ghats are known to have acted as a climatic refuge based on the pollen records (Mishra et al. 2024; Prasad et al. 2009) as well as the persistence of several ancient lineages within the region (Joshi and Karanth 2011, 2013; Gopal et al. 2023; Bharti et al. 2021; Vijayakumar et al. 2016). Overall climatic stability coupled with complex topography supports the persistence of ancient lineages as well as provides opportunities for younger lineages to emerge in the southern Western Ghats, yielding high Phylogenetic Diversity and Endemism in the region. Our findings are consistent with global patterns across taxa where regions with complex topography and historically stable, warm, and wet climates have been shown to harbor higher Species Richness, Phylogenetic Diversity, and Endemism (Wiens and Donoghue 2004; Carnaval et al. 2009; Rahbek, Borregaard, Antonelli, et al. 2019; Paúl et al. 2023; Sandel et al. 2011; Fjeldså et al. 2012).

### 4.4 Caveats

We note that expert‐drawn range maps for birds have been shown to overestimate the actual range of species distributions, particularly at finer scales (Warudkar et al. 2022). This mismatch of distribution may influence the estimates of diversity at finer spatial scales, and caution must be taken while interpreting the results at such scales. This also has an effect on the patterns in endemism recovered, as it is known that endemism patterns are scale‐dependent (Daru et al. 2020). Although our dataset primarily includes forest and woodland‐dependent birds, some widespread species, particularly in groups like Accipitriformes, Strigiformes, and species such as the Rose‐ringed Parakeet *Psittacula krameri* , Alexandrine Parakeet *Psittacula eupatria* , and Spotted Owlet *Athene brama* , are difficult to assign to a single habitat type and may introduce noise. Moreover, habitat classifications were sourced from a global trait database (Tobias et al. 2022), which may not accurately represent habitat preferences for species at regional or local scales. Further, we note that ~15% of the species in this study (29 of 188 species) do not have phylogenetic information in the latest birds of the world phylogeny and have been imputed (McTavish et al. 2025). Several of these species are Western Ghats endemics (Table S4), which may lead to underestimation of diversity metrics for the Western Ghats region, although it may not change much of our current inferences. Lastly, the lack of systematic genetic sampling across peninsular India has been a major limitation in assessing the species limits and uncovering cryptic diversity for several bird species, both in a regional and global context (Reddy 2014).

## 5 Conclusion

In summary, our study provides a comprehensive assessment of taxonomic and phylogenetic diversity patterns in forest birds across peninsular India, revealing strong spatial heterogeneity shaped by a combination of historical, climatic, and topographic factors. The southern Western Ghats emerged as a clear evolutionary hotspot, harboring high species richness, endemism, and an accumulation of ancient lineages. Conversely, regions like the northern Eastern Ghats and central Indian highlands (Satpuras and Chota Nagpur Plateau) exhibited unique assemblages influenced by proximity to the Himalayas and distinct climatic and topographic conditions. Our results also underscored the role of biogeographic barriers, particularly the Goa and Godavari gaps, in shaping community structure and turnover. Importantly, we highlight the influence of climatic stability and complex topography in sustaining high diversity and promoting endemism. While our findings were broadly consistent with patterns observed in other taxa, caveats such as missing phylogenetic data, cryptic diversity, and the use of expert‐drawn range maps underscore the need for finer scale genetic and distributional data. Taken together, this study contributes to our understanding of forest bird diversity in peninsular India and provides critical insights for biogeography in this understudied yet highly biodiverse region and broadly conforms to the global patterns underscoring the importance of historic climatic stability, complex geoclimatic history, and topography in driving patterns in diversity.

## Author Contributions

**Naman Goyal:** conceptualization (lead), data curation (lead), formal analysis (lead), funding acquisition (supporting), investigation (lead), methodology (equal), project administration (equal), software (equal), validation (equal), visualization (lead), writing – original draft (lead), writing – review and editing (equal). **Archita Sharma:** data curation (supporting), formal analysis (supporting), funding acquisition (supporting), investigation (supporting), methodology (equal), software (equal), validation (equal), visualization (supporting), writing – original draft (supporting), writing – review and editing (equal). **Vishwa Jagati:** data curation (supporting), formal analysis (equal), methodology (equal), software (equal), validation (equal). **Akshay Herur:** data curation (supporting), formal analysis (equal), methodology (equal), software (equal), validation (equal). **Arunima Jain:** data curation (supporting), formal analysis (equal), methodology (equal), software (equal), validation (equal). **Abhishek Gopal:** conceptualization (supporting), methodology (equal), software (equal), validation (equal), writing – review and editing (equal). **Jahnavi Joshi:** conceptualization (supporting), methodology (equal), supervision (supporting), validation (equal), writing – review and editing (equal). **V. V. Robin:** conceptualization (supporting), funding acquisition (lead), investigation (supporting), methodology (equal), project administration (equal), resources (lead), supervision (lead), validation (equal), writing – original draft (supporting), writing – review and editing (equal).

## Funding

This work was supported by Rohini Nilekani Philanthropies, G‐202410‐00940.

## Conflicts of Interest

The authors declare no conflicts of interest.

## Supporting information


**Appendix S1:** ece372698‐sup‐0001‐AppendixS1.pdf.

## Data Availability

All raw data, such as bird range maps and occurrence records, are openly available in public repositories cited in the study. The processed data and the codes used in the study have been made available at https://doi.org/10.5061/dryad.6djh9w1fn.

## References

[ece372698-bib-0001] Acevedo, S. , and B. Sandel . 2021. “Phylogenetic Endemism Hotspots of North American Birds Are Associated With Warm Temperatures and Long‐ and Short‐Term Climate Stability.” Frontiers in Ecology and Evolution 9: 396. 10.3389/fevo.2021.645396.

[ece372698-bib-0002] Amatulli, G. , S. Domisch , M.‐N. Tuanmu , et al. 2018. “A Suite of Global, Cross‐Scale Topographic Variables for Environmental and Biodiversity Modeling.” Scientific Data 5, no. 1: 180040. 29557978 10.1038/sdata.2018.40 PMC5859920

[ece372698-bib-0003] Araújo, M. B. , D. Nogués‐Bravo , J. A. F. Diniz‐Filho , A. M. Haywood , P. J. Valdes , and C. Rahbek . 2008. “Quaternary Climate Changes Explain Diversity Among Reptiles and Amphibians.” Ecography 31, no. 1: 8–15.

[ece372698-bib-0004] Aravind, N. A. , K. P. Rajashekhar , and N. A. Madhyastha . 2005. “Species Diversity, Endemism and Distribution of Land Snails of the Western Ghats, India.” Records of the Western Australian Museum Supplement 68, no. 1: 31.

[ece372698-bib-0005] Beck, H. E. , T. R. McVicar , N. Vergopolan , et al. 2023. “High‐Resolution (1 Km) Köppen‐Geiger Maps for 1901–2099 Based on Constrained CMIP6 Projections.” Scientific Data 10, no. 1: 724. 37872197 10.1038/s41597-023-02549-6 PMC10593765

[ece372698-bib-0006] Bharti, D. K. , G. D. Edgecombe , K. P. Karanth , and J. Joshi . 2021. “Spatial Patterns of Phylogenetic Diversity and Endemism in the Western Ghats, India: A Case Study Using Ancient Predatory Arthropods.” Ecology and Evolution 11, no. 23: 16499–16513. 34938452 10.1002/ece3.8119 PMC8668739

[ece372698-bib-0007] BirdLife International, Handbook of the Birds of the World . 2023. “Bird Species Distribution Maps of the World. Version 2023.1.” http://datazone.birdlife.org/species/requestdis.

[ece372698-bib-0008] Biswas, A. , and K. P. Karanth . 2021. “Role of Geographical Gaps in the Western Ghats in Shaping Intra‐ and Interspecific Genetic Diversity.” Journal of the Indian Institute of Science 101, no. 2: 151–164.

[ece372698-bib-0009] Bivand, R. , and G. Piras . 2015. “Comparing Implementations of Estimation Methods for Spatial Econometrics.” Journal of Statistical Software 63, no. 18: 1–36.

[ece372698-bib-0010] Bose, R. , B. R. Ramesh , R. Pélissier , and F. Munoz . 2019. “Phylogenetic Diversity in the Western Ghats Biodiversity Hotspot Reflects Environmental Filtering and Past Niche Diversification of Trees.” Journal of Biogeography 46, no. 1: 145–157.

[ece372698-bib-0011] Carnaval, A. C. , M. J. Hickerson , C. F. B. Haddad , M. T. Rodrigues , and C. Moritz . 2009. “Stability Predicts Genetic Diversity in the Brazilian Atlantic Forest Hotspot.” Science (New York, N.Y.) 323, no. 5915: 785–789. 19197066 10.1126/science.1166955

[ece372698-bib-0012] Cornell, H. V. , and S. P. Harrison . 2014. “What Are Species Pools and When Are They Important?” Annual Review of Ecology, Evolution, and Systematics 45, no. 1: 45–67.

[ece372698-bib-0013] Daniels, R. J. R. 1992. “Geographical Distribution Patterns of Amphibians in the Western Ghats, India.” Journal of Biogeography 19, no. 5: 521.

[ece372698-bib-0014] Daru, B. H. , H. Farooq , A. Antonelli , and S. Faurby . 2020. “Endemism Patterns Are Scale Dependent.” Nature Communications 11, no. 1: 2115. 10.1038/s41467-020-15921-6 PMC7192928 32355257

[ece372698-bib-0015] Davidar, P. , J. P. Puyravaud , and E. G. Leigh Jr. 2005. “Changes in Rain Forest Tree Diversity, Dominance and Rarity Across a Seasonality Gradient in the Western Ghats, India.” Journal of Biogeography 32, no. 3: 493–501.

[ece372698-bib-0016] Dexter, K. G. , R. A. Segovia , and A. R. Griffiths . 2019. “Exploring the Concept of Lineage Diversity Across North American Forests.” Forests 10, no. 6: 520.

[ece372698-bib-0017] Divya, R. , and K. P. Karanth . 2021. “Contrasting Patterns of Phylogenetic Diversity Across Climatic Zones of Western Ghats: A Biodiversity Hotspot in Peninsular India.” Journal of Systematics and Evolution 59, no. 2: 240–250.

[ece372698-bib-0018] Dobson, A. , D. Lodge , J. Alder , et al. 2006. “Habitat Loss, Trophic Collapse, and the Decline of Ecosystem Services.” Ecology 87, no. 8: 1915–1924. 16937628 10.1890/0012-9658(2006)87[1915:hltcat]2.0.co;2

[ece372698-bib-0019] Evanno, G. , S. Regnaut , and J. Goudet . 2005. “Detecting the Number of Clusters of Individuals Using the Software STRUCTURE: A Simulation Study.” Molecular Ecology 14, no. 8: 2611–2620. 15969739 10.1111/j.1365-294X.2005.02553.x

[ece372698-bib-0020] Evans, K. L. , P. H. Warren , and K. J. Gaston . 2005. “Species‐Energy Relationships at the Macroecological Scale: A Review of the Mechanisms.” Biological Reviews of the Cambridge Philosophical Society 80, no. 1: 1–25. 15727036 10.1017/s1464793104006517

[ece372698-bib-0021] Faith, D. P. 1992. “Conservation Evaluation and Phylogenetic Diversity.” Biological Conservation 61, no. 1: 1–10.

[ece372698-bib-0022] Fjeldså, J. , R. C. K. Bowie , and C. Rahbek . 2012. “The Role of Mountain Ranges in the Diversification of Birds.” Annual Review of Ecology, Evolution, and Systematics 43, no. 1: 249–265.

[ece372698-bib-0023] García Molinos, J. , D. S. Schoeman , C. J. Brown , and M. T. Burrows . 2019. “VoCC: An R Package for Calculating the Velocity of Climate Change and Related Climatic Metrics.” Methods in Ecology and Evolution 10, no. 12: 2195–2202.

[ece372698-bib-0024] Giraudoux, P. 2013. “Pgirmess: Data Analysis in Ecology.” R Package Version. https://scholar.google.com/citations?user=R4zO7IYAAAAJ&hl=en&oi=sra.

[ece372698-bib-0025] Gómez, A. , D. H. Lunt , S. Weiss , and N. Ferrand . 2007. “Phylogeography of Southern European Refugia.” In Refugia Within Refugia: Patterns of Phylogeographic Concordance in the Iberian Peninsula, 155–188. Springer.

[ece372698-bib-0026] Gopal, A. , D. K. Bharti , N. Page , et al. 2023. “Range Restricted Old and Young Lineages Show the Southern Western Ghats to be Both a Museum and a Cradle of Diversity for Woody Plants.” Proceedings of the Royal Society B: Biological Sciences 290: 20222513. 10.1098/rspb.2022.2513 PMC10130714 37122248

[ece372698-bib-0027] Gotelli, N. J. , and G. L. Entsminger . 2003. “Swap Algorithms in Null Model Analysis.” Ecology 84, no. 2: 532–535.

[ece372698-bib-0028] Gotelli, N. J. , and W. Ulrich . 2011. “Statistical Challenges in Null Model Analysis.” Oikos 121, no. 2: 171–180. 10.1111/j.1600-0706.2011.20301.x.

[ece372698-bib-0029] Grant, K. R. , T. J. Davies , S. M. Harish , et al. 2023. “Phylogenetic Community Patterns Suggest Central Indian Tropical Dry Forests Are Structured by Montane Climate Refuges.” Diversity and Distributions 29: 13708. 10.1111/ddi.13708.

[ece372698-bib-0030] Hawkins, B. A. , R. Field , H. V. Cornell , et al. 2003. “Energy, Water, and Broad‐Scale Geographic Patterns of Species Richness.” Ecology 84, no. 12: 3105–3117.

[ece372698-bib-0031] Jain, S. , V. Tiwari , A. Thapa , et al. 2022. “Performance Evaluation of Google Earth Engine Based Precipitation Datasets Under Different Climatic Zones Over India.” Remote Sensing in Earth Systems Sciences 5, no. 4: 263–276.

[ece372698-bib-0032] Joshi, J. , and K. P. Karanth . 2011. “Cretaceous‐Tertiary Diversification Among Select Scolopendrid Centipedes of South India.” Molecular Phylogenetics and Evolution 60, no. 3: 287–294. 21575731 10.1016/j.ympev.2011.04.024

[ece372698-bib-0033] Joshi, J. , and P. Karanth . 2013. “Did Southern Western Ghats of Peninsular India Serve as Refugia for Its Endemic Biota During the Cretaceous Volcanism?” Ecology and Evolution 3, no. 10: 3275–3282. 24223267 10.1002/ece3.603 PMC3797476

[ece372698-bib-0034] Karanth, K. 2017. “Species Complex, Species Concepts and Characterization of Cryptic Diversity: Vignettes From Indian Systems.” Current Science 112, no. 7: 1320–1324.

[ece372698-bib-0035] Karger, D. N. , O. Conrad , J. Böhner , et al. 2017. “Climatologies at High Resolution for the Earth's Land Surface Areas.” Scientific Data 4: 170122. 28872642 10.1038/sdata.2017.122 PMC5584396

[ece372698-bib-0036] Karger, D. N. , M. P. Nobis , S. Normand , C. H. Graham , and N. E. Zimmermann . 2023. “CHELSA‐TraCE21k – High‐Resolution (1 Km) Downscaled Transient Temperature and Precipitation Data Since the Last Glacial Maximum.” Climate of the Past 19, no. 2: 439–456.

[ece372698-bib-0037] Kembel, S. W. , P. D. Cowan , M. R. Helmus , et al. 2010. “Picante: R Tools for Integrating Phylogenies and Ecology.” Bioinformatics 26, no. 11: 1463–1464. 20395285 10.1093/bioinformatics/btq166

[ece372698-bib-0038] Kissling, W. D. , and G. Carl . 2008. “Spatial Autocorrelation and the Selection of Simultaneous Autoregressive Models.” Global Ecology and Biogeography: A Journal of Macroecology 17, no. 1: 59–71.

[ece372698-bib-0039] Leprieur, F. , C. Albouy , J. De Bortoli , P. F. Cowman , D. R. Bellwood , and D. Mouillot . 2012. “Quantifying Phylogenetic Beta Diversity: Distinguishing Between ‘True’ Turnover of Lineages and Phylogenetic Diversity Gradients.” PLoS One 7, no. 8: e42760. 22912736 10.1371/journal.pone.0042760 PMC3422232

[ece372698-bib-0040] Loarie, S. R. , P. B. Duffy , H. Hamilton , G. P. Asner , C. B. Field , and D. D. Ackerly . 2009. “The Velocity of Climate Change.” Nature 462, no. 7276: 1052–1055. 20033047 10.1038/nature08649

[ece372698-bib-0041] McTavish, E. J. , J. A. Gerbracht , M. T. Holder , et al. 2025. “A Complete and Dynamic Tree of Birds.” Proceedings of the National Academy of Sciences of the United States of America 122, no. 18: e2409658122. 10.1073/pnas.2409658122. 40299701 PMC12067227

[ece372698-bib-0042] Mishler, B. D. , N. Knerr , C. E. González‐Orozco , A. H. Thornhill , S. W. Laffan , and J. T. Miller . 2014. “Phylogenetic Measures of Biodiversity and Neo‐ and Paleo‐Endemism in Australian Acacia.” Nature Communications 5, no. 1: 4473. 10.1038/ncomms5473 25034856

[ece372698-bib-0043] Mishra, S. , M. Bansal , V. Prasad , et al. 2024. “Did the Deccan Volcanism Impact the Indian Flora During the Maastrichtian?” Earth‐Science Reviews 258, no. 104950: 104950.

[ece372698-bib-0044] Molina, C. , and L. Stone . 2020. “Difficulties in Benchmarking Ecological Null Models: An Assessment of Current Methods.” Ecology 101, no. 3: e02945. 10.1002/ecy.2945. 31834622 PMC7078898

[ece372698-bib-0045] Myers, N. , R. A. Mittermeier , C. G. Mittermeier , G. A. da Fonseca , and J. Kent . 2000. “Biodiversity Hotspots for Conservation Priorities.” Nature 403, no. 6772: 853–858. 10706275 10.1038/35002501

[ece372698-bib-0046] Nitta, J. H. , S. W. Laffan , B. D. Mishler , and W. Iwasaki . 2023. “Canaper: Categorical Analysis of Neo‐ and Paleo‐Endemism in R.” Ecography 2023, no. 9: 638. 10.1111/ecog.06638.

[ece372698-bib-0047] Oksanen, J. , G. L. Simpson , F. G. Blanchet , et al. 2025. “Vegan: Community Ecology Package.” https://vegandevs.github.io/vegan/.

[ece372698-bib-0048] Page, N. V. , and K. Shanker . 2020. “Climatic Stability Drives Latitudinal Trends in Range Size and Richness of Woody Plants in the Western Ghats, India.” PLoS One 15, no. 7: e0235733. 32673330 10.1371/journal.pone.0235733 PMC7365598

[ece372698-bib-0049] Paúl, M. J. , D. Rosauer , P. Tarroso , G. Velo‐Antón , and S. B. Carvalho . 2023. “Environmental and Topographic Drivers of Amphibian Phylogenetic Diversity and Endemism in the Iberian Peninsula.” Ecology and Evolution 13, no. 1: e9666. 36620407 10.1002/ece3.9666 PMC9817204

[ece372698-bib-0050] Pereira, H. M. , P. W. Leadley , V. Proença , et al. 2010. “Scenarios for Global Biodiversity in the 21st Century.” Science (New York, N.Y.) 330, no. 6010: 1496–1501. 20978282 10.1126/science.1196624

[ece372698-bib-0051] Prasad, V. , A. Farooqui , S. K. M. Tripathi , R. Garg , and B. Thakur . 2009. “Evidence of Late Palaeocene‐Early Eocene Equatorial Rain Forest Refugia in Southern Western Ghats, India.” Journal of Biosciences 34, no. 5: 777–797. 20009271 10.1007/s12038-009-0062-y

[ece372698-bib-0052] QGIS Development Team . 2024. QGIS Geographic Information System. QGIS Association.

[ece372698-bib-0053] Rahbek, C. , M. K. Borregaard , A. Antonelli , et al. 2019. “Building Mountain Biodiversity: Geological and Evolutionary Processes.” Science (New York, N.Y.) 365, no. 6458: 1114–1119. 31515384 10.1126/science.aax0151

[ece372698-bib-0054] Rahbek, C. , M. K. Borregaard , R. K. Colwell , et al. 2019. “Humboldt's Enigma: What Causes Global Patterns of Mountain Biodiversity?” Science 365, no. 6458: 1108–1113. 31515383 10.1126/science.aax0149

[ece372698-bib-0055] Ramachandran, V. , V. V. Robin , K. Tamma , and U. Ramakrishnan . 2017. “Climatic and Geographic Barriers Drive Distributional Patterns of Bird Phenotypes Within Peninsular India.” Journal of Avian Biology 48, no. 5: 620–630.

[ece372698-bib-0056] Ramdas, L. A. 1974. “Weather and Climatic Patterns.” In Monographiae Biologicae, 99–134. Springer Netherlands.

[ece372698-bib-0057] Reddy, S. 2014. “What's Missing From Avian Global Diversification Analyses?” Molecular Phylogenetics and Evolution 77: 159–165. 24780750 10.1016/j.ympev.2014.04.023

[ece372698-bib-0058] Ripley, S. D. , and B. M. Beehler . 1990. “Patterns of Speciation in Indian Birds.” Journal of Biogeography 17, no. 6: 639.

[ece372698-bib-0059] Robin, V. V. , C. K. Vishnudas , P. Gupta , and U. Ramakrishnan . 2015. “Deep and Wide Valleys Drive Nested Phylogeographic Patterns Across a Montane Bird Community.” Proceedings of the Royal Society B: Biological Sciences 282, no. 1810: 861. 10.1098/rspb.2015.0861 PMC4590488 26085588

[ece372698-bib-0060] Robin, V. V. , C. K. Vishnudas , P. Gupta , et al. 2017. “Two New Genera of Songbirds Represent Endemic Radiations From the Shola Sky Islands of the Western Ghats, India.” BMC Evolutionary Biology 17, no. 1: 31. 28114902 10.1186/s12862-017-0882-6 PMC5259981

[ece372698-bib-0061] Rosauer, D. , S. W. Laffan , M. D. Crisp , S. C. Donnellan , and L. G. Cook . 2009. “Phylogenetic Endemism: A New Approach for Identifying Geographical Concentrations of Evolutionary History.” Molecular Ecology 18, no. 19: 4061–4072. 19754516 10.1111/j.1365-294X.2009.04311.x

[ece372698-bib-0062] Sandel, B. , L. Arge , B. Dalsgaard , et al. 2011. “The Influence of Late Quaternary Climate‐Change Velocity on Species Endemism.” Science (New York, N.Y.) 334, no. 6056: 660–664. 21979937 10.1126/science.1210173

[ece372698-bib-0063] Strona, G. , D. Nappo , F. Boccacci , S. Fattorini , and J. San‐Miguel‐Ayanz . 2014. “A Fast and Unbiased Procedure to Randomize Ecological Binary Matrices With Fixed Row and Column Totals.” Nature Communications 5, no. 1: 4114. 10.1038/ncomms5114. 24916345

[ece372698-bib-0064] Tobias, J. A. , C. Sheard , A. L. Pigot , et al. 2022. “AVONET: Morphological, Ecological and Geographical Data for All Birds.” Ecology Letters 25, no. 3: 581–597. 35199922 10.1111/ele.13898

[ece372698-bib-0065] Ulrich, W. , and N. J. Gotteli . 2007. “Null Model Analysis of Species Nestedness Patterns.” Ecology 88: 1824–1831. 10.1890/06-1208.1. 17645028

[ece372698-bib-0066] Veron, S. , T. J. Davies , M. W. Cadotte , P. Clergeau , and S. Pavoine . 2017. “Predicting Loss of Evolutionary History: Where Are we?: Predicting Loss of Evolutionary History.” Biological Reviews of the Cambridge Philosophical Society 92, no. 1: 271–291. 26467982 10.1111/brv.12228

[ece372698-bib-0067] Vijayakumar, S. P. , R. C. Menezes , A. Jayarajan , and K. Shanker . 2016. “Glaciations, Gradients, and Geography: Multiple Drivers of Diversification of Bush Frogs in the Western Ghats Escarpment.” Proceedings of the Royal Society B: Biological Sciences 283, no. 1836: 20161011. 10.1098/rspb.2016.1011 PMC5013767 27534957

[ece372698-bib-0068] Vilela, B. , and F. Villalobos . 2015. “letsR: A New R Package for Data Handling and Analysis in Macroecology.” Methods in Ecology and Evolution 6: 12401. 10.1111/2041-210X.12401.

[ece372698-bib-0069] Warudkar, A. , N. Goyal , V. Kher , et al. 2022. “Using the Area of Habitat to Assess the Extent of Protection of India's Birds.” Biotropica 54, no. 6: 1466–1479.

[ece372698-bib-0070] White, A. E. , K. K. Dey , D. Mohan , M. Stephens , and T. Price . 2019. “Regional Influences on Community Structure Across the Tropical‐Temperate Divide.” Nature Communications 10, no. 1: 2646. 10.1038/s41467-019-10253-6 PMC6570764 31201312

[ece372698-bib-0071] White, A. E. , K. K. Dey , M. Stephens , and T. D. Price . 2021. “Dispersal Syndromes Drive the Formation of Biogeographical Regions, Illustrated by the Case of Wallace's Line.” Global Ecology and Biogeography: A Journal of Macroecology 30, no. 3: 685–696. 33776580 10.1111/geb.13250 PMC7986858

[ece372698-bib-0072] Wickham, H. 2016. ggplot2: Elegant Graphics for Data Analysis. 2nd ed. Springer‐Verlag.

[ece372698-bib-0073] Wiens, J. J. , and M. J. Donoghue . 2004. “Historical Biogeography, Ecology and Species Richness.” Trends in Ecology & Evolution 19, no. 12: 639–644. 16701326 10.1016/j.tree.2004.09.011

[ece372698-bib-0074] Wilson, A. M. , and W. Jetz . 2016. “Remotely Sensed High‐Resolution Global Cloud Dynamics for Predicting Ecosystem and Biodiversity Distributions.” PLoS Biology 14, no. 3: e1002415. 27031693 10.1371/journal.pbio.1002415 PMC4816575

