# Supplementary material for: Spatial Patterns of Diversity in Forest Birds of Peninsular India
Source: Ecol Evol. 2025 Dec 16;15(12):e72698. doi: 10.1002/ece3.72698 (PMC12706521; doi:10.1002/ece3.72698)
Supplement: Supplementary file 1 — Appendix S1: ece372698‐sup‐0001‐AppendixS1.pdf. [file ECE3-15-e72698-s001.pdf]

Supplementary Information

Figure S1 Phylogeny of the 188 species of forest and woodland habitat birds selected in this study

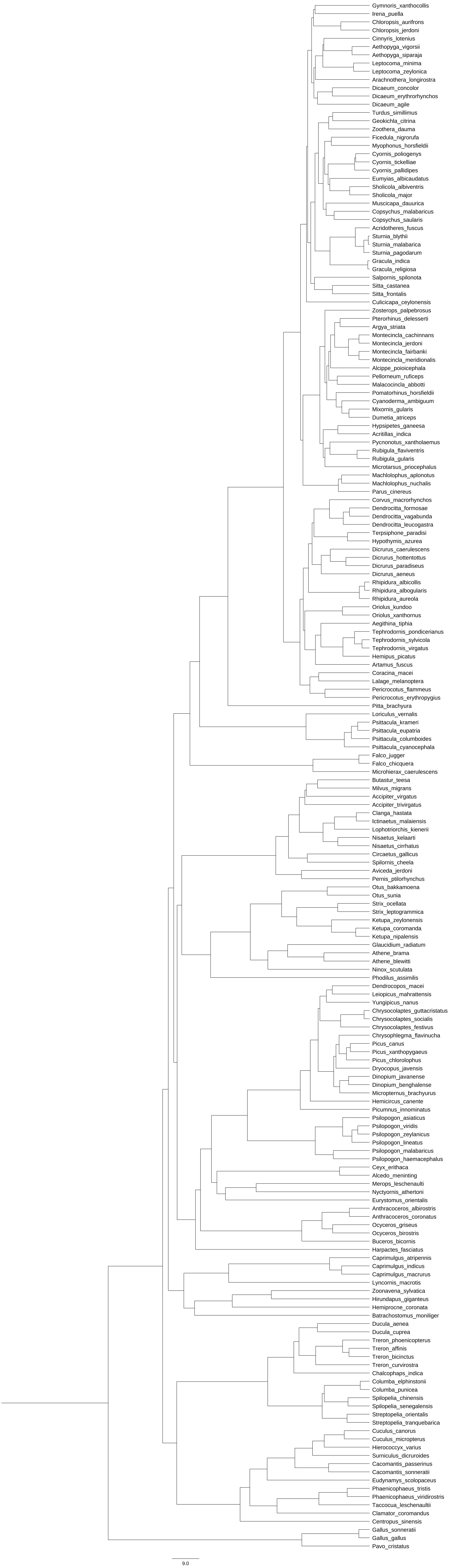
Gymnoris_xanthocollis
Irena_puella
Chloropsis_aurifrons
Chloropsis_jerdoni
Cinnyris_lotenius
Aethopyga_vigorsii
Aethopyga_siparaja
Leptocoma_minima
Leptocoma_zeylonica
Arachnothera_longirostra
Dicaeum_concolor
Dicaeum_erythrorhynchos
Dicaeum_agile
Turdus_simillimus
Geokichla_citrina
Zoothera_dauma
Ficedula_nigrorufa
Myophonus_horsfieldii
Cyornis_poliogenys
Cyornis_tickelliae
Cyornis_pallidipes
Eumyias_albicaudatus
Sholicola_albiventris
Sholicola_major
Muscicapa_dauurica
Copsychus_malabaricus
Copsychus_saularis
Acridotheres_fuscus
Sturnia_blythii
Sturnia_malabarica
Sturnia_pagodarum
Gracula_indica
Gracula_religiosa
Salpornis_spilonota
Sitta_castanea
Sitta_frontalis
Culicicapa_ceylonensis
Zosterops_palpebrosus
Pterorhinus_delesserti
Argya_striata
Montecincla_cachinnans
Montecincla_jerdoni
Montecincla_fairbanki
Montecincla_meridionalis
Alcippe_poioicephala
Pellorneum_ruficeps
Malacocincla_abbotti
Pomatorhinus_horsfieldii
Cyanoderma_ambiguum
Mixornis_gularis
Dumetia_atriceps
Hypsipetes_ganeesa
Acritillas_indica
Pycnonotus_xantholaemus
Rubigula_flaviventris
Rubigula_gularis
Microtarsus_priocephalus
Machlolophus_aplonotus
Machlolophus_nuchalis
Parus_cinereus
Corvus_macrorhynchos
Dendrocitta_formosae
Dendrocitta_vagabunda
Dendrocitta_leucogastra
Terpsiphone_paradisi
Hypothymis_azurea
Dicrurus_caerulescens
Dicrurus_hottentottus
Dicrurus_paradiseus
Dicrurus_aeneus
Rhipidura_albicollis
Rhipidura_albogularis
Rhipidura_aureola
Oriolus_kundoo
Oriolus_xanthornus
Aegithina_tiphia
Tephrodornis_pondicerianus
Tephrodornis_sylvicola
Tephrodornis_virgatus
Hemipus_picatus
Artamus_fuscus
Coracina_macei
Lalage_melanoptera
Pericrocotus_flammeus
Pericrocotus_erythropygius
Pitta_brachyura
Loriculus_vernalis
Psittacula_krameri
Psittacula_eupatria
Psittacula_columboides
Psittacula_cyanocephala
Falco_jugger
Falco_chicquera
Microhierax_caerulescens
Butastur_teesa
Milvus_migrans
Accipiter_virgatus
Accipiter_trivirgatus
Clanga_hastata
Ictinaetus_malaiensis
Lophotriorchis_kienerii
Nisaetus_kelaarti
Nisaetus_cirrhatus
Circaetus_gallicus
Spilornis_cheela
Aviceda_jerdoni
Pernis_ptilorhynchus
Otus_bakkamoena
Otus_sunia
Strix_ocellata
Strix_leptogrammica
Ketupa_zeylonensis
Ketupa_coromanda
Ketupa_nipalensis
Glaucidium_radiatum
Athene_brama
Athene_blewitti
Ninox_scutulata
Phodilus_assimilis
Dendrocopos_macei
Leiopicus_mahrattensis
Yungipicus_nanus
Chrysocolaptes_guttacristatus
Chrysocolaptes_socialis
Chrysocolaptes_festivus
Chrysophlegma_flavinucha
Picus_canus
Picus_xanthopygaeus
Picus_chlorolophus
Dryocopus_javensis
Dinopium_javanense
Dinopium_benghalense
Micropternus_brachyurus
Hemicircus_canente
Picumnus_innominatus
Psilopogon_asiaticus
Psilopogon_viridis
Psilopogon_zeylanicus
Psilopogon_lineatus
Psilopogon_malabaricus
Psilopogon_haemacephalus
Ceyx_erithaca
Alcedo_meninting
Merops_leschenaulti
Nyctyornis_athertoni
Eurystomus_orientalis
Anthracoceros_albirostris
Anthracoceros_coronatus
Ocyceros_griseus
Ocyceros_birostris
Buceros_bicornis
Harpactes_fasciatus
Caprimulgus_atripennis
Caprimulgus_indicus
Caprimulgus_macrurus
Lyncornis_macrotis
Zoonavena_sylvatica
Hirundapus_giganteus
Hemiprocne_coronata
Batrachostomus_moniliger
Ducula_aenea
Ducula_cuprea
Treron_phoenicopterus
Treron_affinis
Treron_bicinctus
Treron_curvirostra
Chalcophaps_indica
Columba_elphinstonii
Columba_punicea
Spilopelia_chinensis
Spilopelia_senegalensis
Streptopelia_orientalis
Streptopelia_tranquebarica
Cuculus_canorus
Cuculus_micropterus
Hierococcyx_varius
Surniculus_dicruroides
Cacomantis_passerinus
Cacomantis_sonneratii
Eudynamys_scolopaceus
Phaenicophaeus_tristis
Phaenicophaeus_viridirostris
Taccocua_leschenaultii
Clamator_coromandus
Centropus_sinensis
Gallus_sonneratii
Gallus_gallus
Pavo_cristatus
9.0

Figure S2 Optimization for *n_reps* at A) 10km x 10km and B) 25km x 25km scales. Here, pd_obs_p_upper is the percentile of observed phylogenetic diversity relative to random. We compare the values between two independent runs and select the n_reps at which the two runs start converging.

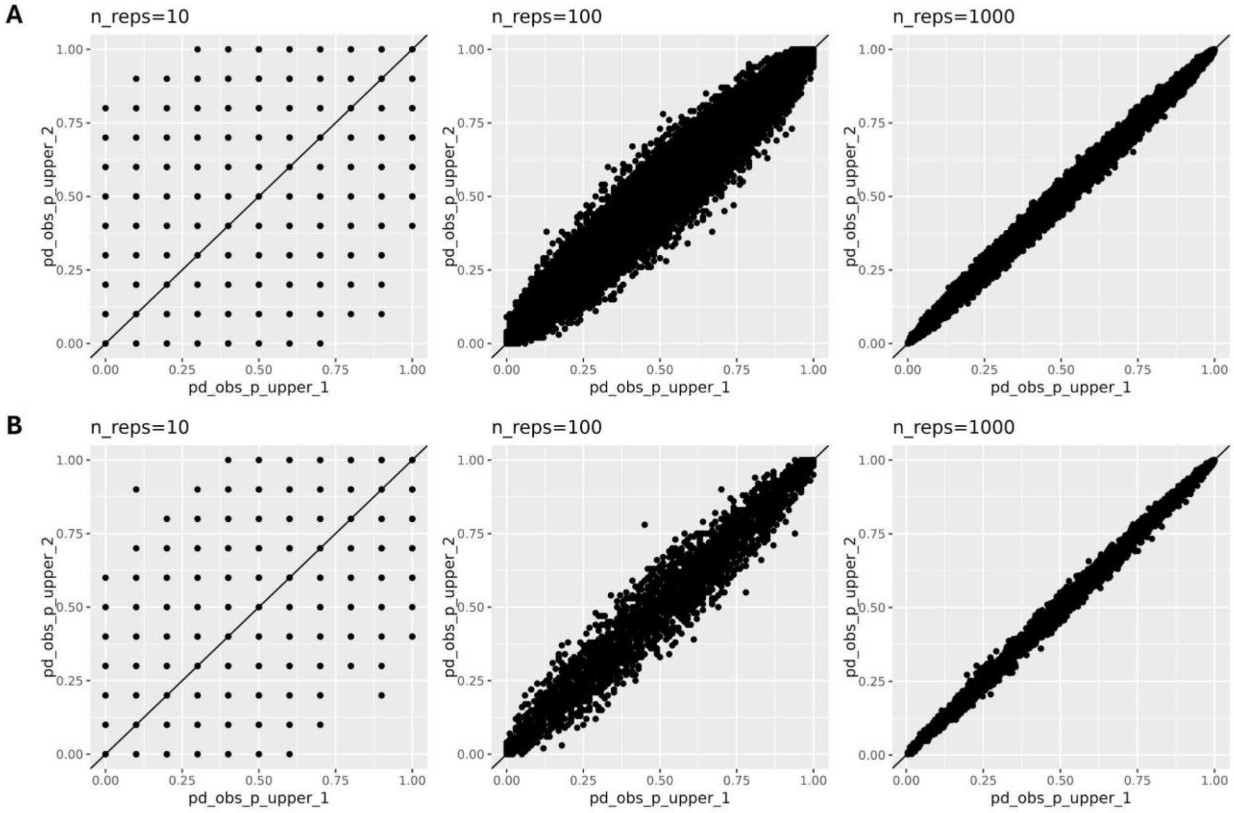

Figure S3 Optimization for *n_iterations* at A) 10km x 10km and B) 25km x 25km scales

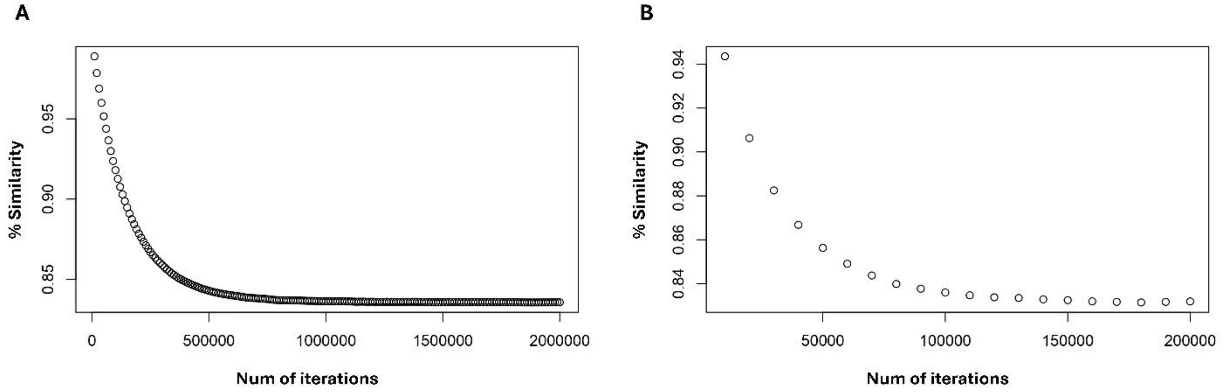

Figure S4 Linear regression analysis examining relationships between mapped variables (25km x25 km scale for birdlife distribution data). (Abbreviations on the plot - spdv = Species Richness, pd_obs = Phylogenetic Diversity, pe_obs = Phylogenetic Endemism, rpd_obs = Relative Phylogenetic Diversity, and rpe_obs = Relative Phylogenetic Endemism). Linear regression analysis examining relationships between PD, PE, RPD, and RPD shows that they are significantly correlated, although there is some scatter in certain relationships.

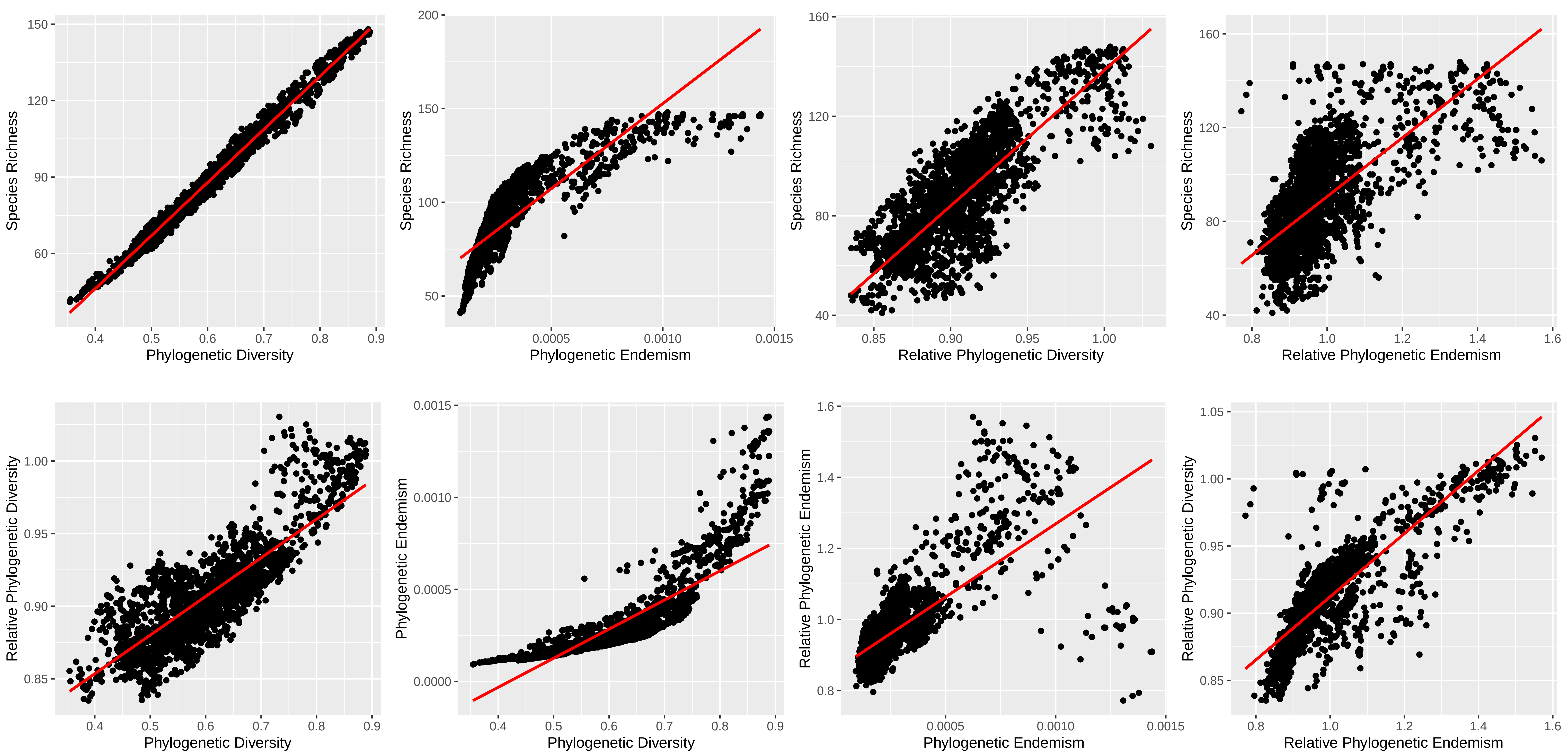
Species Richness
Phylogenetic Diversity
Phylogenetic Endemism
Relative Phylogenetic Diversity
Relative Phylogenetic Endemism
Relative Phylogenetic Diversity
Phylogenetic Diversity
Phylogenetic Endemism
Phylogenetic Diversity
Relative Phylogenetic Endemism
Phylogenetic Endemism
Relative Phylogenetic Diversity
Relative Phylogenetic Endemism

Figure S5 Patterns in diversity of forest birds in peninsular India at 10x10km scale using BirdLife International range maps, A) Species richness, B) Weighted Endemism (WE), C) Phylogenetic Diversity (PD), D) Phylogenetic Endemism (PE), E) Relative Phylogenetic Diversity (RPD), F) Relative Phylogenetic Endemism (RPE)

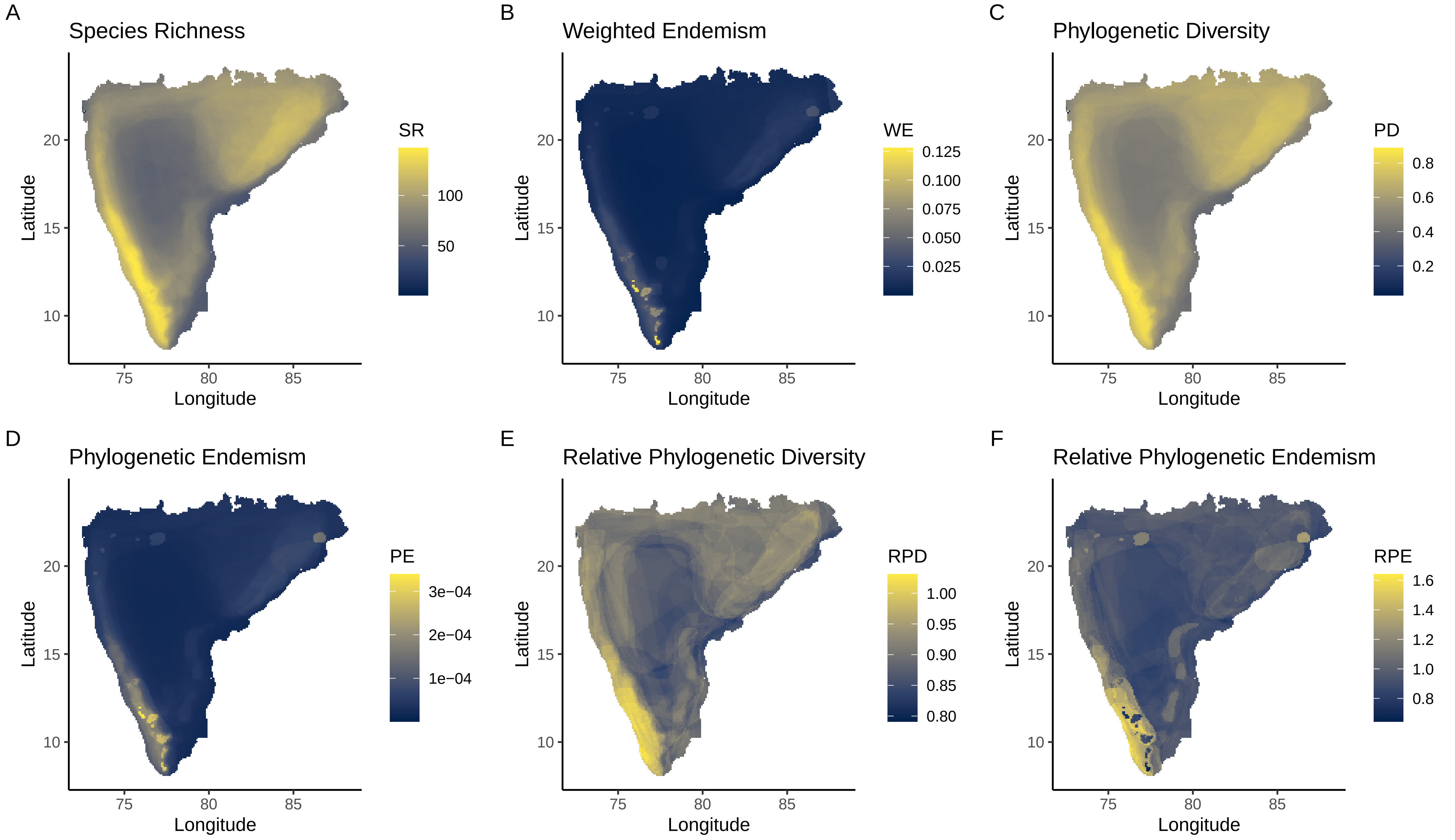
A
Species Richness
SR
100
50
B
Weighted Endemism
WE
0.125
0.100
0.075
0.050
0.025
C
Phylogenetic Diversity
PD
0.8
0.6
0.4
0.2
D
Phylogenetic Endemism
PE
3e−04
2e−04
1e−04
E
Relative Phylogenetic Diversity
RPD
1.00
0.95
0.90
0.85
0.80
F
Relative Phylogenetic Endemism
RPE
1.6
1.4
1.2
1.0
0.8
Latitude
20
15
10
Longitude
75
80
85

Figure S6 Patterns in diversity of forest birds in peninsular India at 50x50km scale using BirdLife International range maps, A) Species richness, B) Weighted Endemism (WE), C) Phylogenetic Diversity (PD), D) Phylogenetic Endemism (PE), E) Relative Phylogenetic Diversity (RPD), F) Relative Phylogenetic Endemism (RPE)

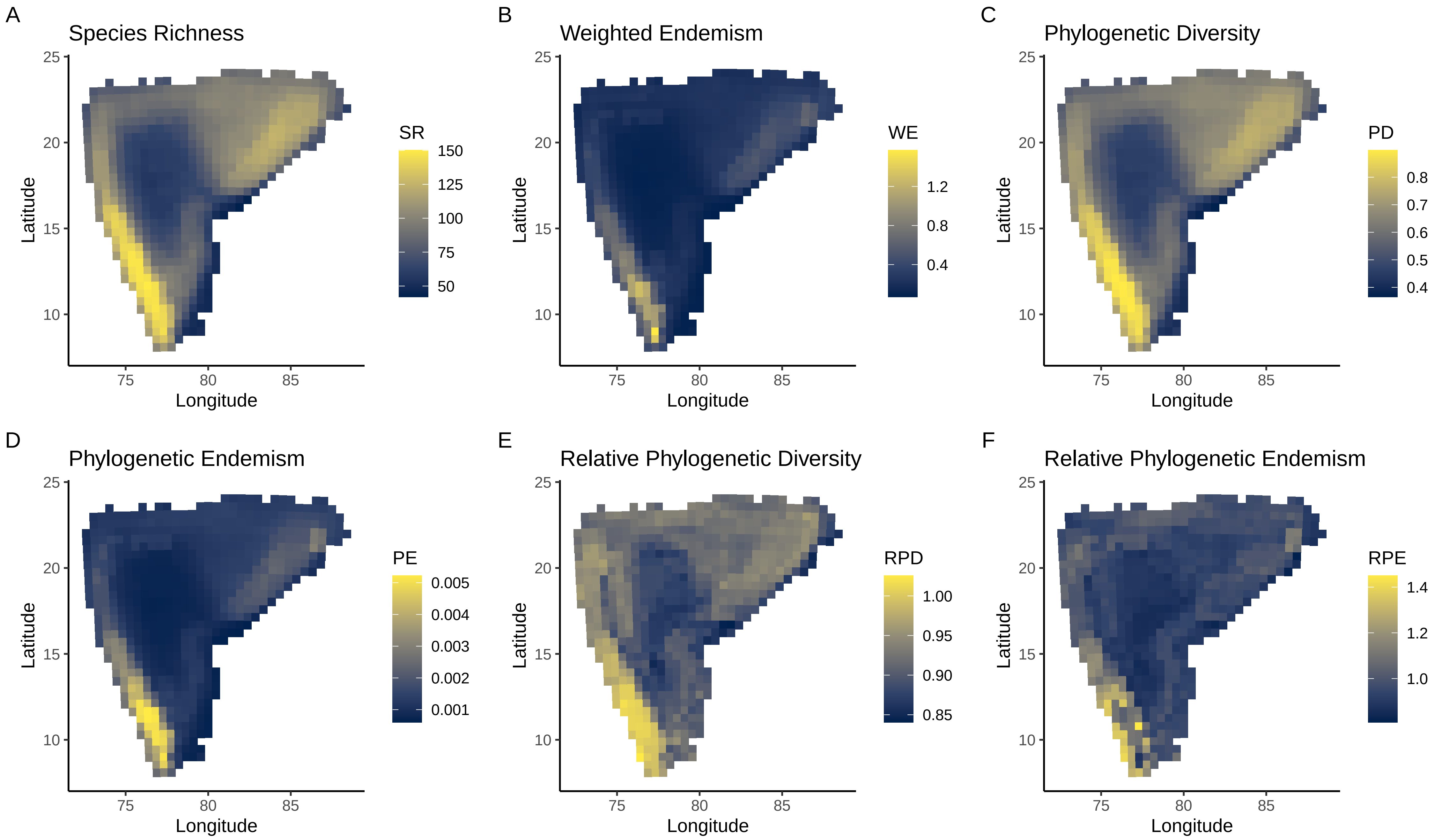
A
Species Richness
SR
150
125
100
75
50
B
Weighted Endemism
WE
1.2
0.8
0.4
C
Phylogenetic Diversity
PD
0.8
0.7
0.6
0.5
0.4
D
Phylogenetic Endemism
PE
0.005
0.004
0.003
0.002
0.001
E
Relative Phylogenetic Diversity
RPD
1.00
0.95
0.90
0.85
F
Relative Phylogenetic Endemism
RPE
1.4
1.2
1.0
Latitude
Longitude
25
20
15
10
75
80
85

Figure S7 Time-integrated Lineage Diversity plots for peninsular India at three different scales - A) 50km x 50km, B) 25km x 25km, C) 10km x10km.

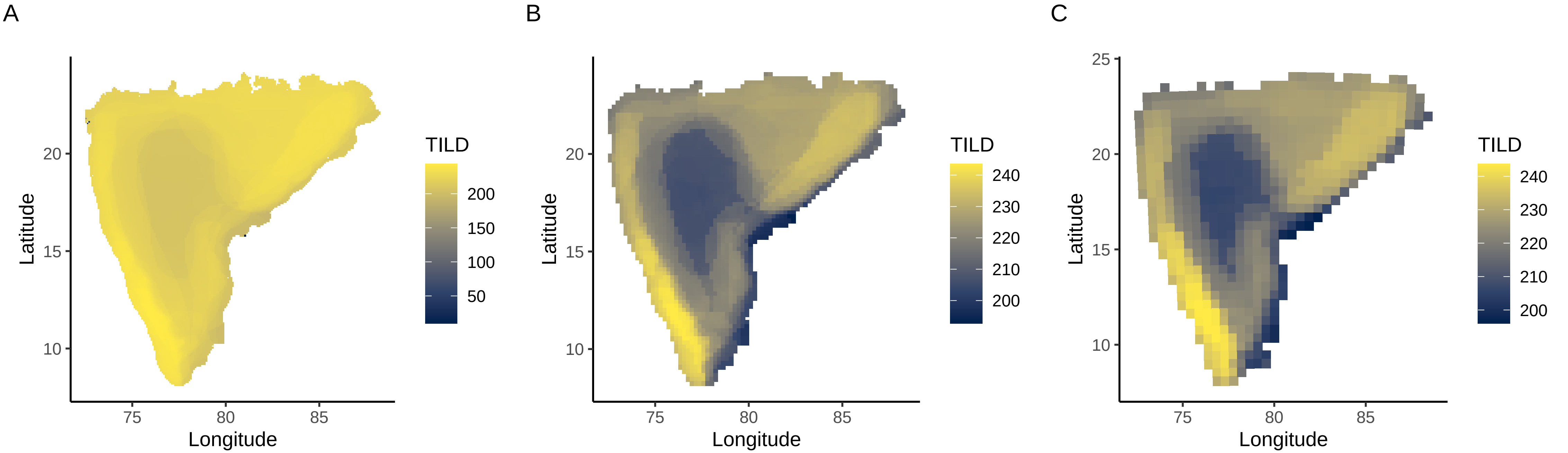

A
B
C
TILD
200
150
100
50
TILD
240
230
220
210
200
TILD
240
230
220
210
200
Latitude
Longitude
25
20
15
10
75
80
85

Figure S8 Patterns in diversity of forest birds in peninsular India at 25x25km scale using eBird data, A) Species Richness, B) Weighted Endemism, C) Phylogenetic Diversity (PD), D) Phylogenetic Endemism (PE), E) Relative Phylogenetic Diversity (RPD), F) Relative Phylogenetic Endemism (RPE). Brighter colours depict areas with high values and darker colours depict areas with low values.

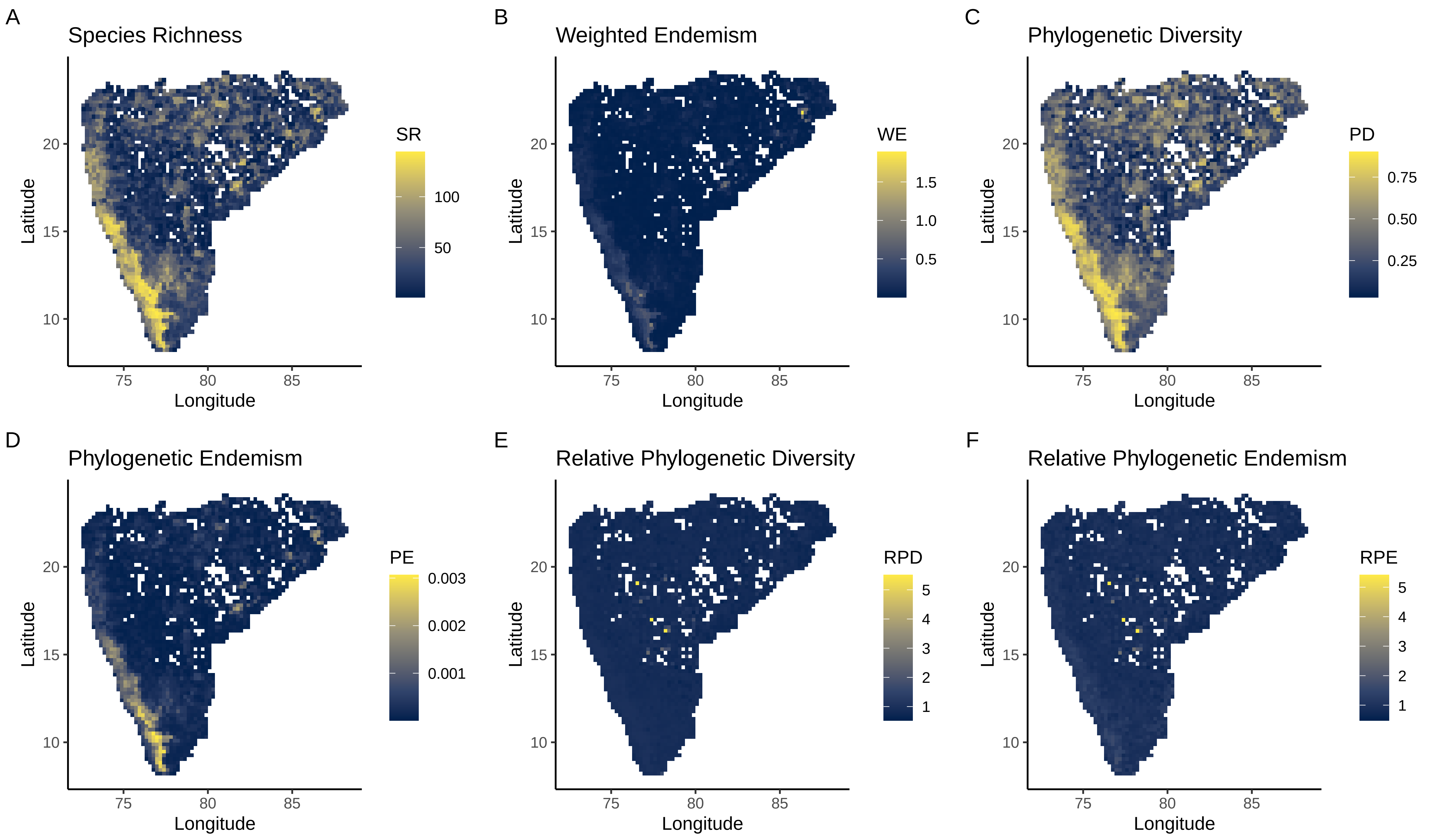

A
Species Richness
SR
100
50
B
Weighted Endemism
WE
1.5
1.0
0.5
C
Phylogenetic Diversity
PD
0.75
0.50
0.25
D
Phylogenetic Endemism
PE
0.003
0.002
0.001
E
Relative Phylogenetic Diversity
RPD
5
4
3
2
1
F
Relative Phylogenetic Endemism
RPE
5
4
3
2
1
Latitude
Longitude
10
15
20
75
80
85

Figure S9 Endemism Type at three different scales - A) 10km x 10km, B) 25km x 25km, and C) 50km x 50km. Endemism type is classified as Mixed, Neo, Paleo, Super and Non-Signficant based on the method defined in Nitta et al. 2023. Mixed - high proportion of both young and old lineages, Neo - significantly high proportion of young lineages, Paleo - significantly high proportion of old lineages, Super - significantly high proportion of both young and old lineages.

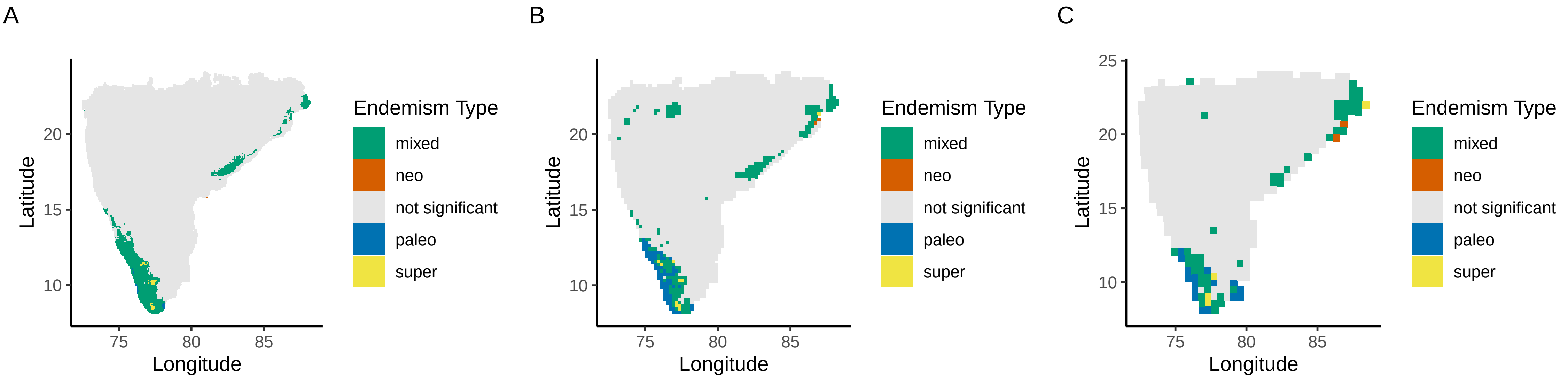

A
B
C
Latitude
Longitude
25
20
15
10
75
80
85
Endemism Type
mixed
neo
not significant
paleo
super

Figure S10 Elbow plot depicting the optimal value of K at 5 for log likelihood for the best model for each K. The log likelihood curve tends to start flattening out after K at 5.

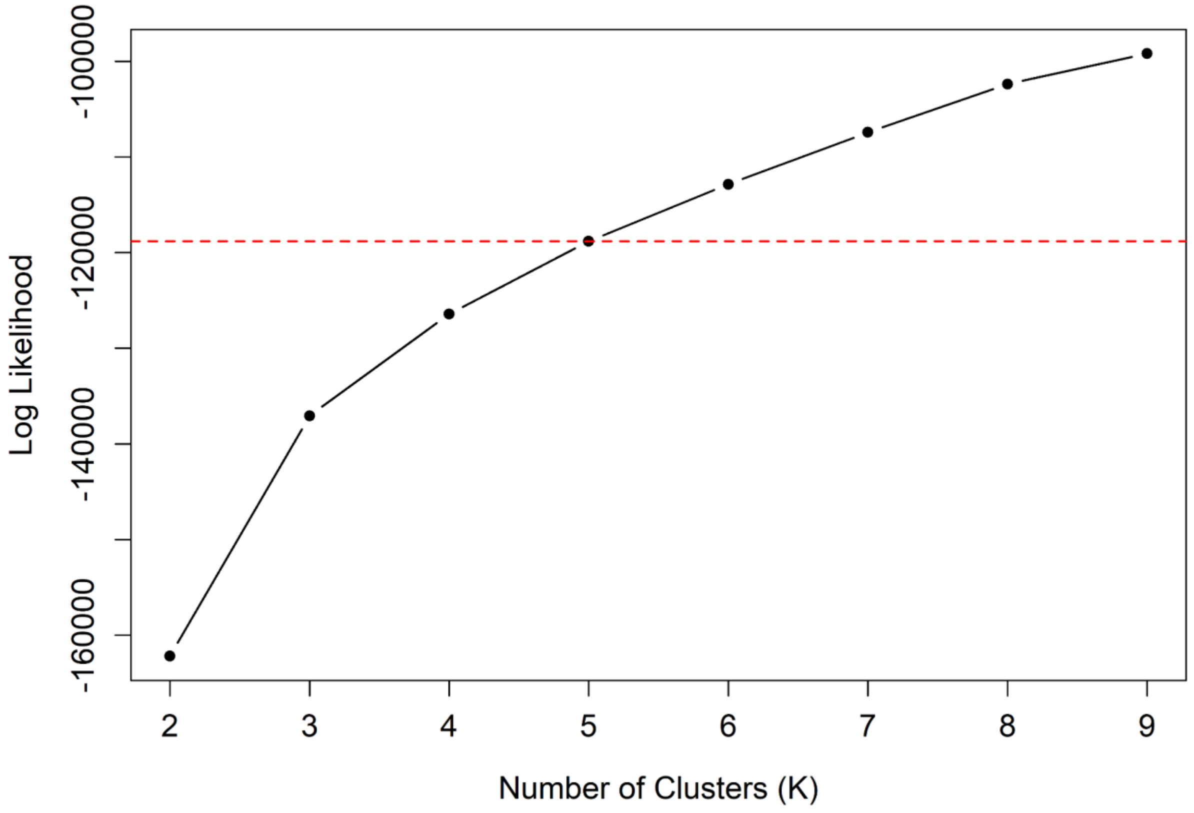

Figure S11 Elbow plot depicting the optimal value of K at 4 for K-means clustering of Sorenson's phylogenetic beta diversity after which the within-cluster sum of squares (WSS) does not decrease significantly.

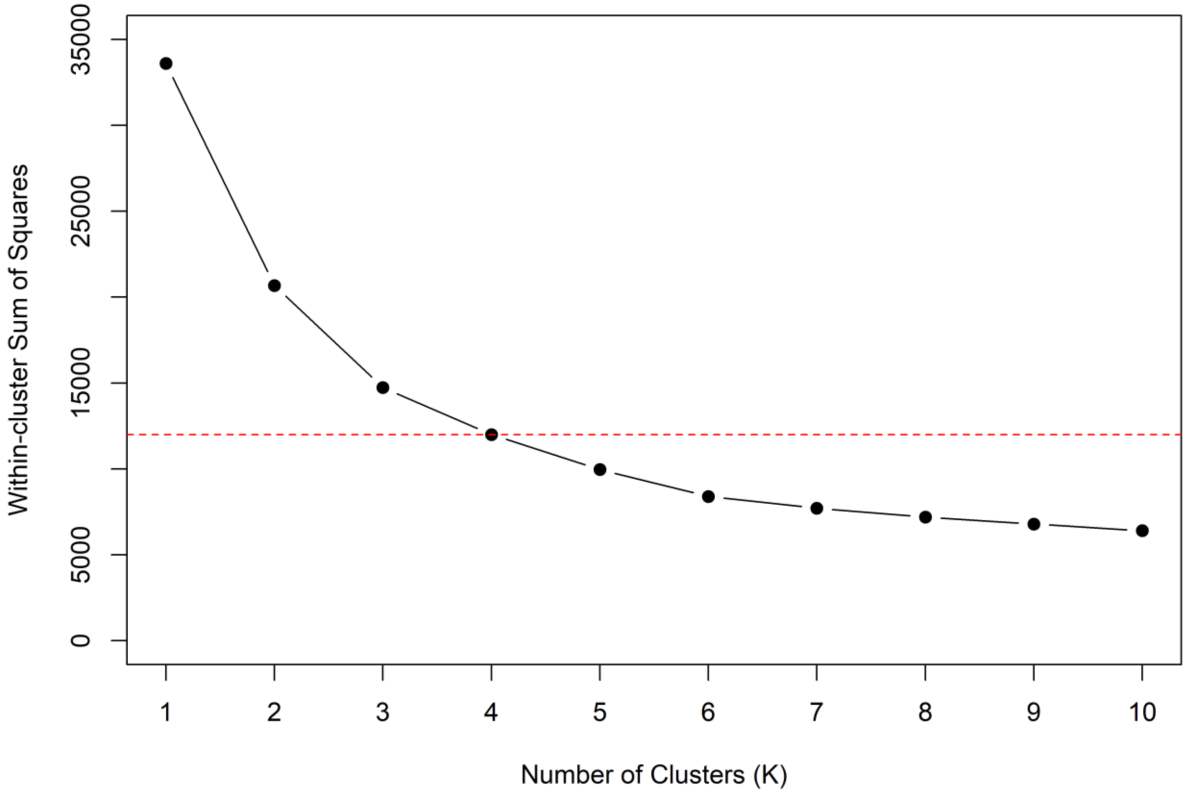

Figure S12 Patterns in community assemblage of forest birds based on Grade of Membership models fitted on species presence-absence data for values of K ranging from 2 to 9. Here each pie chart on a map represents a grid, and the colours depict the proportion of membership derived from distinct communities based on values of K.

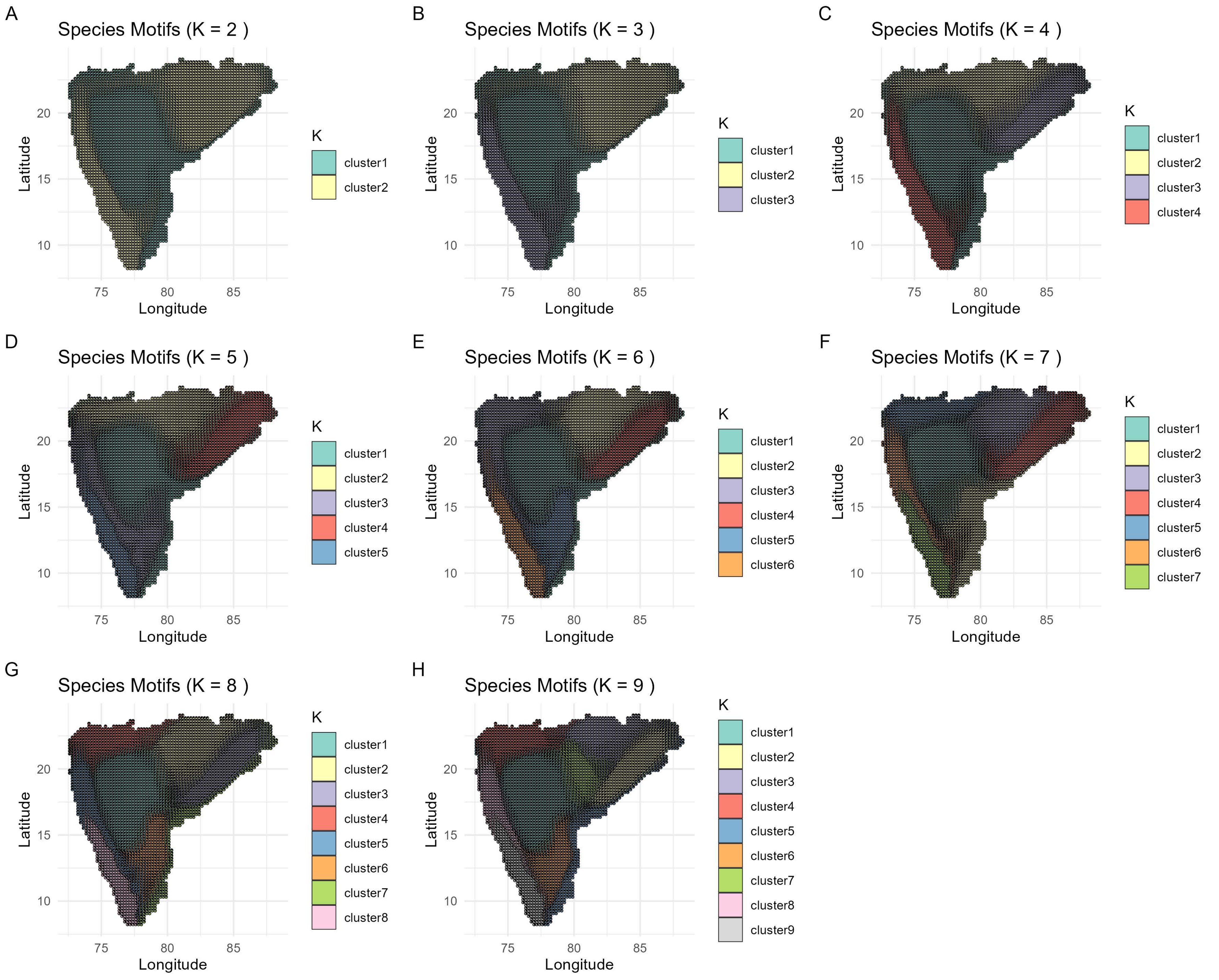
A
Species Motifs (K = 2)
B
Species Motifs (K = 3)
C
Species Motifs (K = 4)
D
Species Motifs (K = 5)
E
Species Motifs (K = 6)
F
Species Motifs (K = 7)
G
Species Motifs (K = 8)
H
Species Motifs (K = 9)
Latitude
Longitude
K
cluster1
cluster2
cluster3
cluster4
cluster5
cluster6
cluster7
cluster8
cluster9
10
15
20
75
80
85

Figure S13 Patterns in Sorenson's Phylogenetic beta diversity across peninsular India using K-means clustering algorithm for values of K from 2 to 9. Here each color depicts a cluster of phylogenetically similar regions.

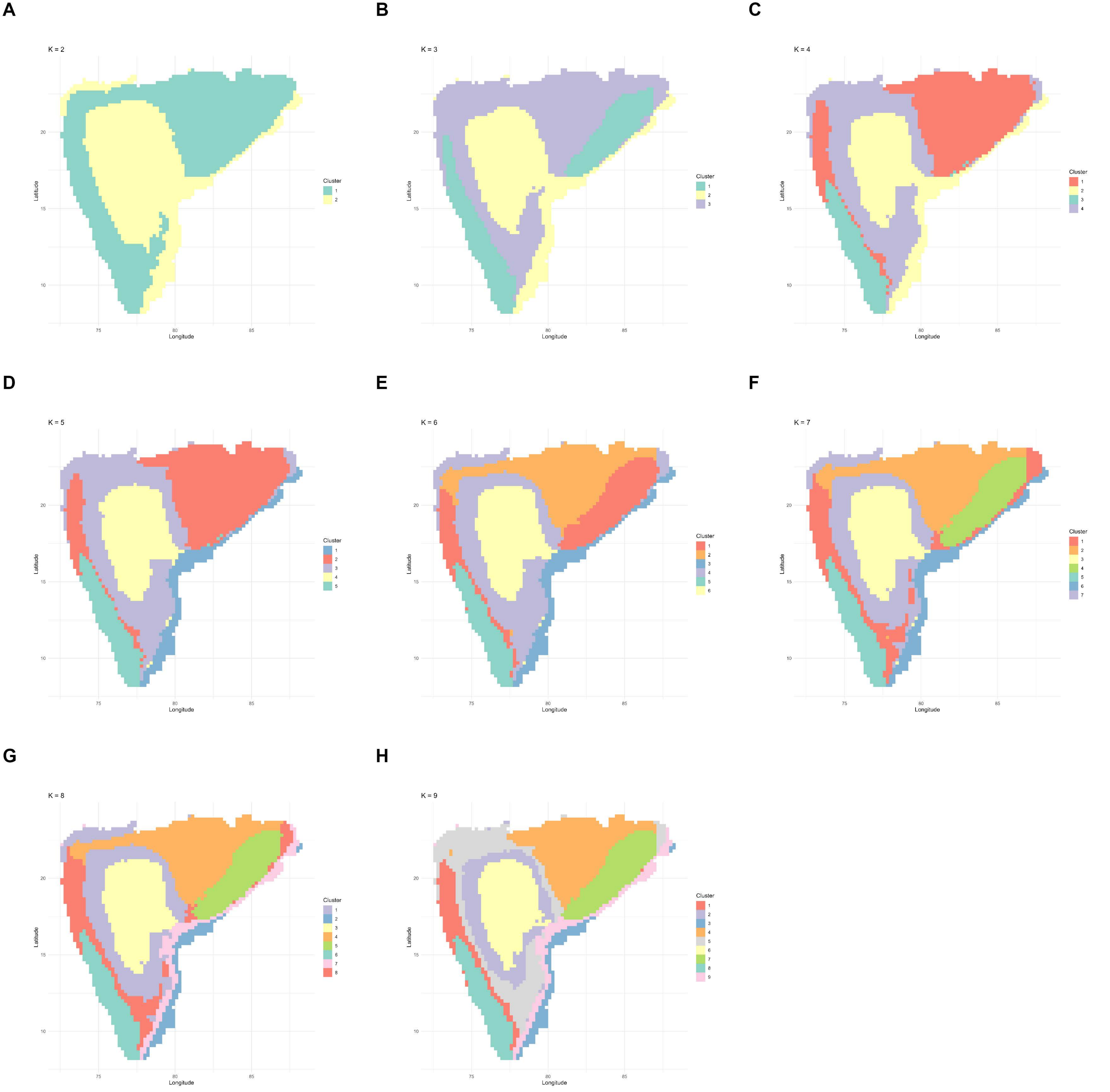
A
K = 2
Cluster
1
2
B
K = 3
Cluster
1
2
3
C
K = 4
Cluster
1
2
3
4
D
K = 5
Cluster
1
2
3
4
5
E
K = 6
Cluster
1
2
3
4
5
6
F
K = 7
Cluster
1
2
3
4
5
6
7
G
K = 8
Cluster
1
2
3
4
5
6
7
8
H
K = 9
Cluster
1
2
3
4
5
6
7
8
9
Latitude
10
15
20
Longitude
75
80
85

Figure S14 Patterns in Simpson's Phylogenetic beta diversity across peninsular India using K-means clustering algorithm for values of K from 2 to 9. Here each color depicts a cluster of phylogenetically similar regions.

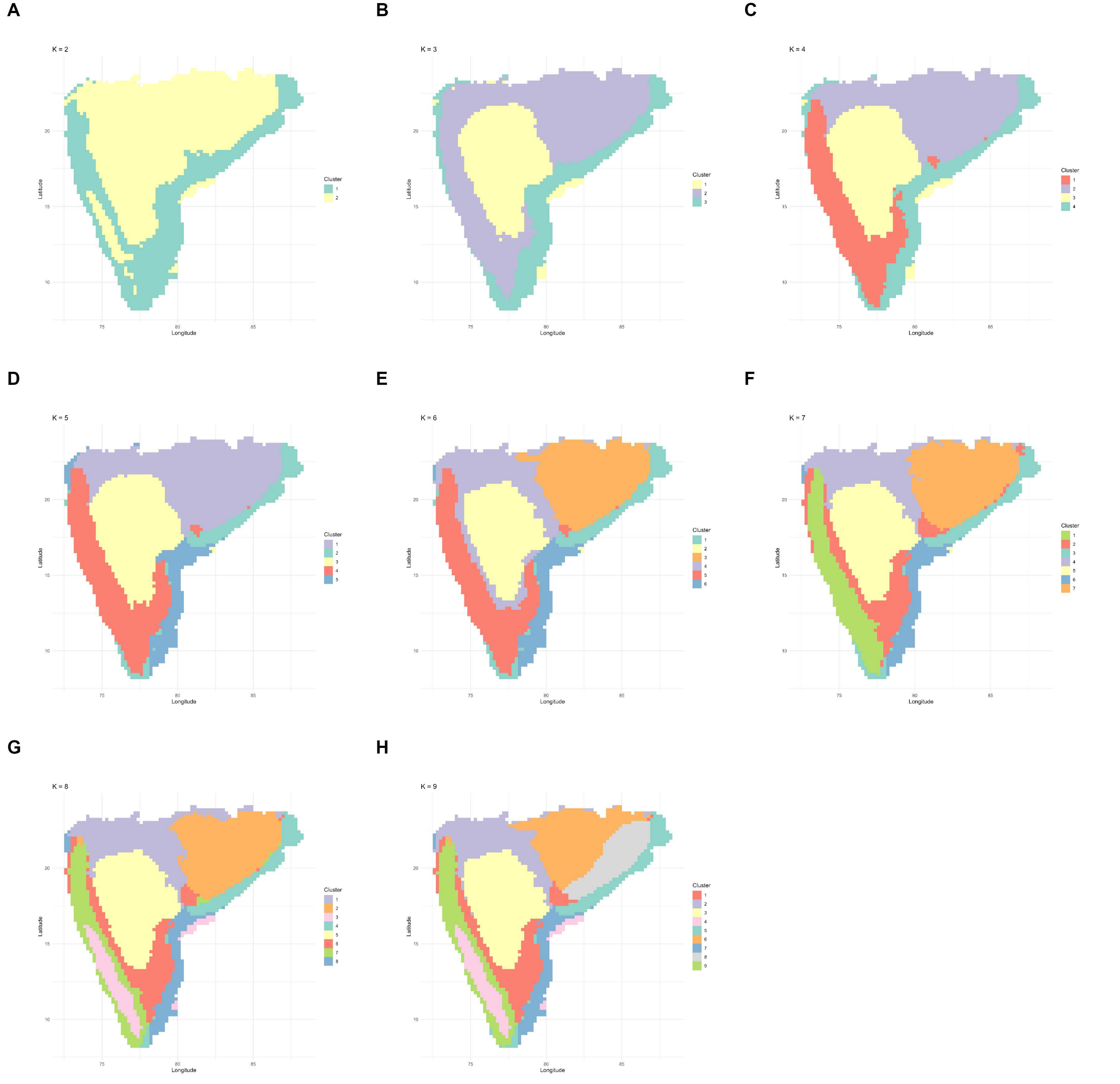
A
K = 2
B
K = 3
C
K = 4
D
K = 5
E
K = 6
F
K = 7
G
K = 8
H
K = 9
Latitude
Longitude
75
80
85
10
15
20
Cluster
1
2
3
4
5
6
7
8
9

Figure S15 Estimates of Relative Phylogenetic Diversity and Relative Phylogenetic Endemism using “Swap” (A and D), “Curveball” (B and E), and “r0” (C and F) algorithms for null models.

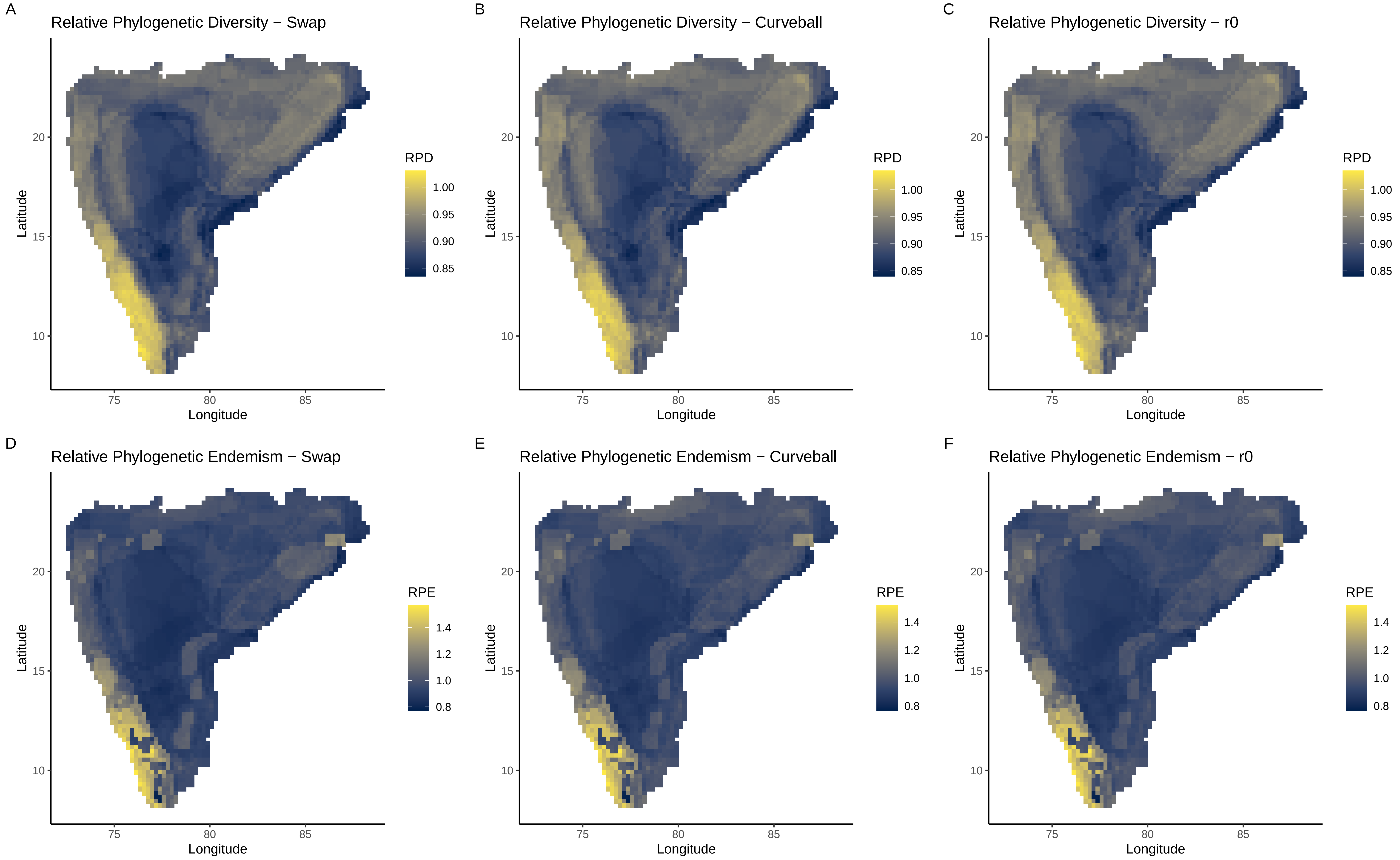
A
Relative Phylogenetic Diversity − Swap
B
Relative Phylogenetic Diversity − Curveball
C
Relative Phylogenetic Diversity − r0
D
Relative Phylogenetic Endemism − Swap
E
Relative Phylogenetic Endemism − Curveball
F
Relative Phylogenetic Endemism − r0
Latitude
Longitude
10
15
20
75
80
85
RPD
1.00
0.95
0.90
0.85
RPE
1.4
1.2
1.0
0.8

Figure S16 Classification of endemism type using A) “Swap”, B) “Curveball”, and C) “r0” algorithms for null models.

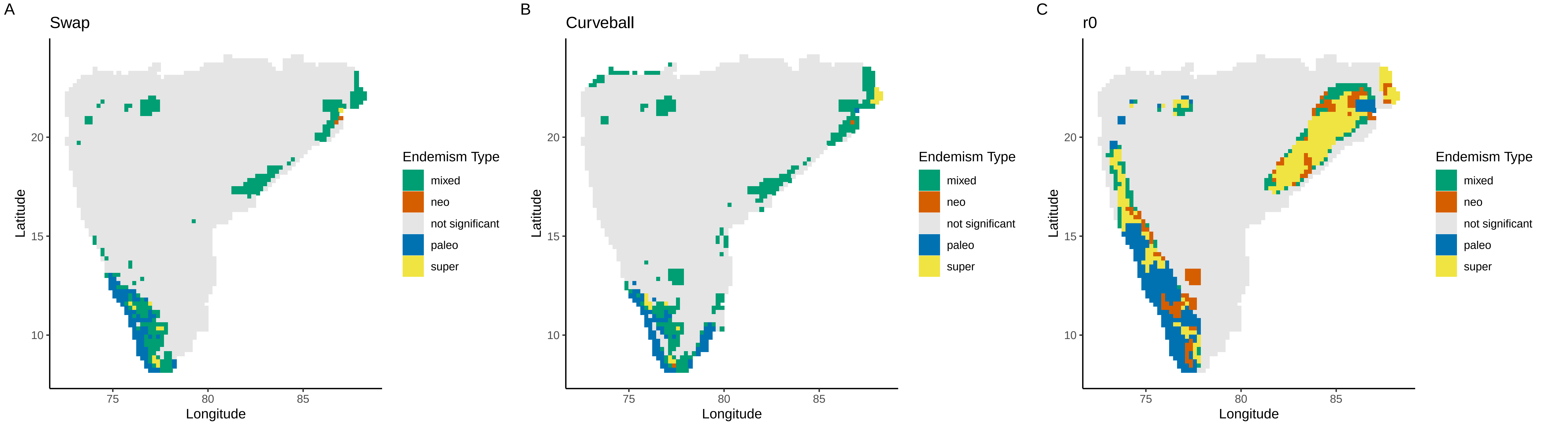
A
Swap
B
Curveball
C
r0
Latitude
Longitude
10
15
20
75
80
85
Endemism Type
mixed
neo
not significant
paleo
super

Figure S17 Significance of Relative Phylogenetic Diversity (A-C), and Relative Phylogenetic Endemism (D-F), for three different null models – “*swap*”, “*curveball*”, and “*r0*”.

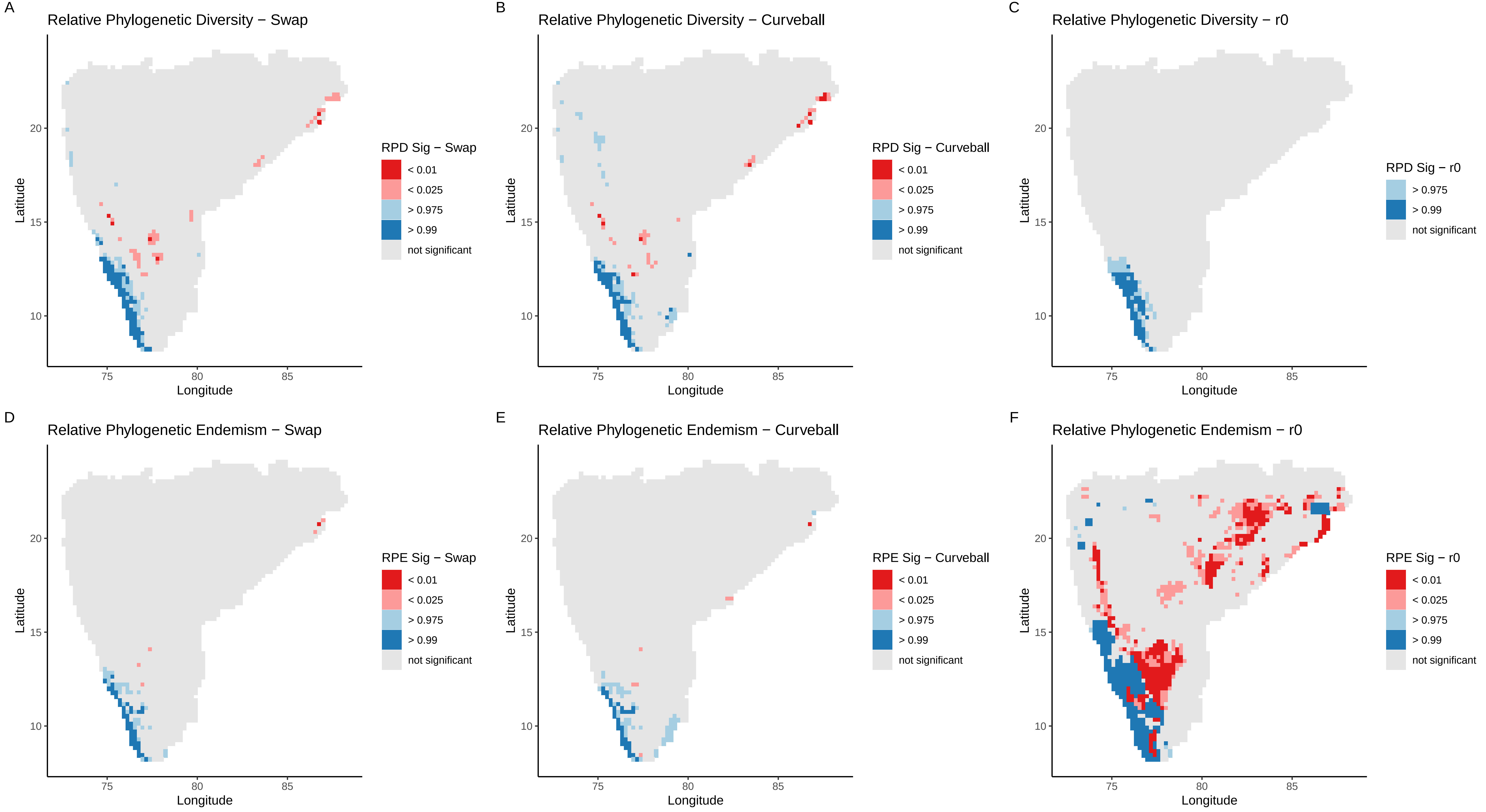
A
Relative Phylogenetic Diversity − Swap
RPD Sig − Swap
< 0.01
< 0.025
> 0.975
> 0.99
not significant
B
Relative Phylogenetic Diversity − Curveball
RPD Sig − Curveball
< 0.01
< 0.025
> 0.975
> 0.99
not significant
C
Relative Phylogenetic Diversity − r0
RPD Sig − r0
> 0.975
> 0.99
not significant
D
Relative Phylogenetic Endemism − Swap
RPE Sig − Swap
< 0.01
< 0.025
> 0.975
> 0.99
not significant
E
Relative Phylogenetic Endemism − Curveball
RPE Sig − Curveball
< 0.01
< 0.025
> 0.975
> 0.99
not significant
F
Relative Phylogenetic Endemism − r0
RPE Sig − r0
< 0.01
< 0.025
> 0.975
> 0.99
not significant
Latitude
Longitude
10
15
20
75
80
85

Table S1 List of resident breeding birds of forest and woodland habitats in peninsular India based on Avonet data (Tobias et al. 2022)

Sno	Species	Order	Family
1	*Accipiter trivirgatus*	Accipitriformes	Accipitridae (Hawks, Eagles, and Kites)
2	*Accipiter virgatus*	Accipitriformes	Accipitridae (Hawks, Eagles, and Kites)
3	*Aviceda jerdoni*	Accipitriformes	Accipitridae (Hawks, Eagles, and Kites)
4	*Butastur teesa*	Accipitriformes	Accipitridae (Hawks, Eagles, and Kites)
5	*Circaetus gallicus*	Accipitriformes	Accipitridae (Hawks, Eagles, and Kites)
6	*Clanga hastata*	Accipitriformes	Accipitridae (Hawks, Eagles, and Kites)
7	*Ictinaetus malaiensis*	Accipitriformes	Accipitridae (Hawks, Eagles, and Kites)
8	*Lophotriorchis kienerii*	Accipitriformes	Accipitridae (Hawks, Eagles, and Kites)
9	*Milvus migrans*	Accipitriformes	Accipitridae (Hawks, Eagles, and Kites)
10	*Nisaetus cirrhatus*	Accipitriformes	Accipitridae (Hawks, Eagles, and Kites)
11	*Nisaetus kelaarti*	Accipitriformes	Accipitridae (Hawks, Eagles, and Kites)
12	*Pernis ptilorhynchus*	Accipitriformes	Accipitridae (Hawks, Eagles, and Kites)
13	*Spilornis cheela*	Accipitriformes	Accipitridae (Hawks, Eagles, and Kites)
14	*Anthracoceros albirostris*	Bucerotiformes	Bucerotidae (Hornbills)
15	*Anthracoceros coronatus*	Bucerotiformes	Bucerotidae (Hornbills)
16	*Buceros bicornis*	Bucerotiformes	Bucerotidae (Hornbills)

17	*Ocyceros birostris*	Bucerotiformes	Bucerotidae (Hornbills)
18	*Ocyceros griseus*	Bucerotiformes	Bucerotidae (Hornbills)
19	*Hirundapus giganteus*	Caprimulgiformes	Apodidae (Swifts)
20	*Zoonavena sylvatica*	Caprimulgiformes	Apodidae (Swifts)
21	*Caprimulgus atripennis*	Caprimulgiformes	Caprimulgidae (Nightjars and Allies)
22	*Caprimulgus indicus*	Caprimulgiformes	Caprimulgidae (Nightjars and Allies)
23	*Caprimulgus macrurus*	Caprimulgiformes	Caprimulgidae (Nightjars and Allies)
24	*Lyncornis macrotis*	Caprimulgiformes	Caprimulgidae (Nightjars and Allies)
25	*Hemiprocne coronata*	Caprimulgiformes	Hemiprocnidae (Treeswifts)
26	*Batrachostomus moniliger*	Caprimulgiformes	Podargidae (Frogmouths)
27	*Chalcophaps indica*	Columbiformes	Columbidae (Pigeons and Doves)
28	*Columba elphinstonii*	Columbiformes	Columbidae (Pigeons and Doves)
29	*Columba punicea*	Columbiformes	Columbidae (Pigeons and Doves)
30	*Ducula aenea*	Columbiformes	Columbidae (Pigeons and Doves)
31	*Ducula cuprea*	Columbiformes	Columbidae (Pigeons and Doves)
32	*Spilopelia chinensis*	Columbiformes	Columbidae (Pigeons and Doves)
33	*Spilopelia senegalensis*	Columbiformes	Columbidae (Pigeons and Doves)

34	*Streptopelia orientalis*	Columbiformes	Columbidae (Pigeons and Doves)
35	*Streptopelia tranquebarica*	Columbiformes	Columbidae (Pigeons and Doves)
36	*Treron affinis*	Columbiformes	Columbidae (Pigeons and Doves)
37	*Treron bicinctus*	Columbiformes	Columbidae (Pigeons and Doves)
38	*Treron curvirostra*	Columbiformes	Columbidae (Pigeons and Doves)
39	*Treron phoenicopterus*	Columbiformes	Columbidae (Pigeons and Doves)
40	*Alcedo meninting*	Coraciiformes	Alcedinidae (Kingfishers)
41	*Ceyx erithaca*	Coraciiformes	Alcedinidae (Kingfishers)
42	*Eurystomus orientalis*	Coraciiformes	Coraciidae (Rollers)
43	*Merops leschenaulti*	Coraciiformes	Meropidae (Bee-eaters)
44	*Nyctyornis athertoni*	Coraciiformes	Meropidae (Bee-eaters)
45	*Cacomantis passerinus*	Cuculiformes	Cuculidae (Cuckoos)
46	*Cacomantis sonneratii*	Cuculiformes	Cuculidae (Cuckoos)
47	*Centropus sinensis*	Cuculiformes	Cuculidae (Cuckoos)
48	*Clamator coromandus*	Cuculiformes	Cuculidae (Cuckoos)
49	*Cuculus canorus*	Cuculiformes	Cuculidae (Cuckoos)
50	*Cuculus micropterus*	Cuculiformes	Cuculidae (Cuckoos)
51	*Eudynamys scolopaceus*	Cuculiformes	Cuculidae (Cuckoos)

52	*Hierococcyx varius*	Cuculiformes	Cuculidae (Cuckoos)
53	*Phaenicophaeus tristis*	Cuculiformes	Cuculidae (Cuckoos)
54	*Phaenicophaeus viridirostris*	Cuculiformes	Cuculidae (Cuckoos)
55	*Surniculus dicruroides*	Cuculiformes	Cuculidae (Cuckoos)
56	*Taccocua leschenaultii*	Cuculiformes	Cuculidae (Cuckoos)
57	*Falco chicquera*	Falconiformes	Falconidae (Falcons and Caracaras)
58	*Falco jugger*	Falconiformes	Falconidae (Falcons and Caracaras)
59	*Microhierax caerulescens*	Falconiformes	Falconidae (Falcons and Caracaras)
60	*Gallus gallus*	Galliformes	Phasianidae (Pheasants, Grouse, and Allies)
61	*Gallus sonneratii*	Galliformes	Phasianidae (Pheasants, Grouse, and Allies)
62	*Pavo cristatus*	Galliformes	Phasianidae (Pheasants, Grouse, and Allies)
63	*Aegithina tiphia*	Passeriformes	Aegithinidae (Ioras)
64	*Artamus fuscus*	Passeriformes	Artamidae (Woodswallows, Bellmagpies, and Allies)
65	*Coracina macei*	Passeriformes	Campephagidae (Cuckooshrikes)
66	*Lalage melanoptera*	Passeriformes	Campephagidae (Cuckooshrikes)
67	*Pericrocotus erythropygius*	Passeriformes	Campephagidae (Cuckooshrikes)
68	*Pericrocotus flammeus*	Passeriformes	Campephagidae (Cuckooshrikes)

69	*Salpornis spilonota*	Passeriformes	Certhiidae (Treecreepers)
70	*Chloropsis aurifrons*	Passeriformes	Chloropseidae (Leafbirds)
71	*Chloropsis jerdoni*	Passeriformes	Chloropseidae (Leafbirds)
72	*Corvus macrorhynchos*	Passeriformes	Corvidae (Crows, Jays, and Magpies)
73	*Dendrocitta formosae*	Passeriformes	Corvidae (Crows, Jays, and Magpies)
74	*Dendrocitta leucogastra*	Passeriformes	Corvidae (Crows, Jays, and Magpies)
75	*Dendrocitta vagabunda*	Passeriformes	Corvidae (Crows, Jays, and Magpies)
76	*Dicaeum agile*	Passeriformes	Dicaeidae (Flowerpeckers)
77	*Dicaeum concolor*	Passeriformes	Dicaeidae (Flowerpeckers)
78	*Dicaeum erythrorhynchos*	Passeriformes	Dicaeidae (Flowerpeckers)
79	*Dicrurus aeneus*	Passeriformes	Dicruridae (Drongos)
80	*Dicrurus caerulescens*	Passeriformes	Dicruridae (Drongos)
81	*Dicrurus hottentottus*	Passeriformes	Dicruridae (Drongos)
82	*Dicrurus paradiseus*	Passeriformes	Dicruridae (Drongos)
83	*Irena puella*	Passeriformes	Irenidae (Fairy-bluebirds)
84	*Alcippe poioicephala*	Passeriformes	Leiothrichidae (Laughingthrushes and Allies)
85	*Argya striata*	Passeriformes	Leiothrichidae (Laughingthrushes and Allies)
86	*Montecincla cachinnans*	Passeriformes	Leiothrichidae (Laughingthrushes and Allies)

87	*Montecincla fairbanki*	Passeriformes	Leiothrichidae (Laughingthrushes and Allies)
88	*Montecincla jerdoni*	Passeriformes	Leiothrichidae (Laughingthrushes and Allies)
89	*Montecincla meridionalis*	Passeriformes	Leiothrichidae (Laughingthrushes and Allies)
90	*Pterorhinus delesserti*	Passeriformes	Leiothrichidae (Laughingthrushes and Allies)
91	*Hypothymis azurea*	Passeriformes	Monarchidae (Monarch Flycatchers)
92	*Terpsiphone paradisi*	Passeriformes	Monarchidae (Monarch Flycatchers)
93	*Copsychus malabaricus*	Passeriformes	Muscicapidae (Old World Flycatchers)
94	*Copsychus saularis*	Passeriformes	Muscicapidae (Old World Flycatchers)
95	*Cyornis pallidipes*	Passeriformes	Muscicapidae (Old World Flycatchers)
96	*Cyornis poliogenys*	Passeriformes	Muscicapidae (Old World Flycatchers)
97	*Cyornis tickelliae*	Passeriformes	Muscicapidae (Old World Flycatchers)
98	*Eumyias albicaudatus*	Passeriformes	Muscicapidae (Old World Flycatchers)
99	*Ficedula nigrorufa*	Passeriformes	Muscicapidae (Old World Flycatchers)
100	*Muscicapa dauurica*	Passeriformes	Muscicapidae (Old World Flycatchers)
101	*Myophonus horsfieldii*	Passeriformes	Muscicapidae (Old World Flycatchers)
102	*Sholicola albiventris*	Passeriformes	Muscicapidae (Old World Flycatchers)
103	*Sholicola major*	Passeriformes	Muscicapidae (Old World Flycatchers)

104	*Aethopyga siparaja*	Passeriformes	Nectariniidae (Sunbirds and Spiderhunters)
105	*Aethopyga vigorsii*	Passeriformes	Nectariniidae (Sunbirds and Spiderhunters)
106	*Arachnothera longirostra*	Passeriformes	Nectariniidae (Sunbirds and Spiderhunters)
107	*Cinnyris lotenius*	Passeriformes	Nectariniidae (Sunbirds and Spiderhunters)
108	*Leptocoma minima*	Passeriformes	Nectariniidae (Sunbirds and Spiderhunters)
109	*Leptocoma zeylonica*	Passeriformes	Nectariniidae (Sunbirds and Spiderhunters)
110	*Oriolus kundoo*	Passeriformes	Oriolidae (Old World Orioles)
111	*Oriolus xanthornus*	Passeriformes	Oriolidae (Old World Orioles)
112	*Machlolophus aplonotus*	Passeriformes	Paridae (Tits, Chickadees, and Titmice)
113	*Machlolophus nuchalis*	Passeriformes	Paridae (Tits, Chickadees, and Titmice)
114	*Parus cinereus*	Passeriformes	Paridae (Tits, Chickadees, and Titmice)
115	*Gymnoris xanthocollis*	Passeriformes	Passeridae (Old World Sparrows)
116	*Malacocincla abbotti*	Passeriformes	Pellorneidae (Ground Babblers and Allies)
117	*Pellorneum ruficeps*	Passeriformes	Pellorneidae (Ground Babblers and Allies)
118	*Pitta brachyura*	Passeriformes	Pittidae (Pittas)
119	*Acritillas indica*	Passeriformes	Pycnonotidae (Bulbuls)
120	*Hypsipetes ganeesa*	Passeriformes	Pycnonotidae (Bulbuls)

121	*Microtarsus priocephalus*	Passeriformes	Pycnonotidae (Bulbuls)
122	*Pycnonotus xantholaemus*	Passeriformes	Pycnonotidae (Bulbuls)
123	*Rubigula flaviventris*	Passeriformes	Pycnonotidae (Bulbuls)
124	*Rubigula gularis*	Passeriformes	Pycnonotidae (Bulbuls)
125	*Rhipidura albicollis*	Passeriformes	Rhipiduridae (Fantails)
126	*Rhipidura albogularis*	Passeriformes	Rhipiduridae (Fantails)
127	*Rhipidura aureola*	Passeriformes	Rhipiduridae (Fantails)
128	*Sitta castanea*	Passeriformes	Sittidae (Nuthatches)
129	*Sitta frontalis*	Passeriformes	Sittidae (Nuthatches)
130	*Culicicapa ceylonensis*	Passeriformes	Stenostiridae (Fairy Flycatchers)
131	*Acridotheres fuscus*	Passeriformes	Sturnidae (Starlings)
132	*Gracula indica*	Passeriformes	Sturnidae (Starlings)
133	*Gracula religiosa*	Passeriformes	Sturnidae (Starlings)
134	*Sturnia blythii*	Passeriformes	Sturnidae (Starlings)
135	*Sturnia malabarica*	Passeriformes	Sturnidae (Starlings)
136	*Sturnia pagodarum*	Passeriformes	Sturnidae (Starlings)
137	*Cyanoderma ambiguum*	Passeriformes	Timaliidae (Tree-Babblers, Scimitar-Babblers, and Allies)

138	*Dumetia atriceps*	Passeriformes	Timaliidae (Tree-Babblers, Scimitar-Babblers, and Allies)
139	*Mixornis gularis*	Passeriformes	Timaliidae (Tree-Babblers, Scimitar-Babblers, and Allies)
140	*Pomatorhinus horsfieldii*	Passeriformes	Timaliidae (Tree-Babblers, Scimitar-Babblers, and Allies)
141	*Geokichla citrina*	Passeriformes	Turdidae (Thrushes and Allies)
142	*Turdus simillimus*	Passeriformes	Turdidae (Thrushes and Allies)
143	*Zoothera dauma*	Passeriformes	Turdidae (Thrushes and Allies)
144	*Hemipus picatus*	Passeriformes	Vangidae (Vangas, Helmetshrikes, and Allies)
145	*Tephrodornis pondicerianus*	Passeriformes	Vangidae (Vangas, Helmetshrikes, and Allies)
146	*Tephrodornis sylvicola*	Passeriformes	Vangidae (Vangas, Helmetshrikes, and Allies)
147	*Tephrodornis virgatus*	Passeriformes	Vangidae (Vangas, Helmetshrikes, and Allies)
148	*Zosterops palpebrosus*	Passeriformes	Zosteropidae (White-eyes, Yuhinas, and Allies)
149	*Psilopogon asiaticus*	Piciformes	Megalaimidae (Asian Barbets)
150	*Psilopogon haemacephalus*	Piciformes	Megalaimidae (Asian Barbets)
151	*Psilopogon lineatus*	Piciformes	Megalaimidae (Asian Barbets)
152	*Psilopogon malabaricus*	Piciformes	Megalaimidae (Asian Barbets)
153	*Psilopogon viridis*	Piciformes	Megalaimidae (Asian Barbets)

154	*Psilopogon zeylanicus*	Piciformes	Megalaimidae (Asian Barbets)
155	*Chrysocolaptes festivus*	Piciformes	Picidae (Woodpeckers)
156	*Chrysocolaptes guttacristatus*	Piciformes	Picidae (Woodpeckers)
157	*Chrysocolaptes socialis*	Piciformes	Picidae (Woodpeckers)
158	*Chrysophlegma flavinucha*	Piciformes	Picidae (Woodpeckers)
159	*Dendrocopos macei*	Piciformes	Picidae (Woodpeckers)
160	*Dinopium benghalense*	Piciformes	Picidae (Woodpeckers)
161	*Dinopium javanense*	Piciformes	Picidae (Woodpeckers)
162	*Dryocopus javensis*	Piciformes	Picidae (Woodpeckers)
163	*Hemicircus canente*	Piciformes	Picidae (Woodpeckers)
164	*Leiopicus mahrattensis*	Piciformes	Picidae (Woodpeckers)
165	*Micropternus brachyurus*	Piciformes	Picidae (Woodpeckers)
166	*Picumnus innominatus*	Piciformes	Picidae (Woodpeckers)
167	*Picus canus*	Piciformes	Picidae (Woodpeckers)
168	*Picus chlorolophus*	Piciformes	Picidae (Woodpeckers)
169	*Picus xanthopygaeus*	Piciformes	Picidae (Woodpeckers)
170	*Yungipicus nanus*	Piciformes	Picidae (Woodpeckers)
171	*Loriculus vernalis*	Psittaciformes	Psittaculidae (Old World Parrots)

172	*Psittacula columboides*	Psittaciformes	Psittaculidae (Old World Parrots)
173	*Psittacula cyanocephala*	Psittaciformes	Psittaculidae (Old World Parrots)
174	*Psittacula eupatria*	Psittaciformes	Psittaculidae (Old World Parrots)
175	*Psittacula krameri*	Psittaciformes	Psittaculidae (Old World Parrots)
176	*Athene blewitti*	Strigiformes	Strigidae (Owls)
177	*Athene brama*	Strigiformes	Strigidae (Owls)
178	*Glaucidium radiatum*	Strigiformes	Strigidae (Owls)
179	*Ketupa coromanda*	Strigiformes	Strigidae (Owls)
180	*Ketupa nipalensis*	Strigiformes	Strigidae (Owls)
181	*Ketupa zeylonensis*	Strigiformes	Strigidae (Owls)
182	*Ninox scutulata*	Strigiformes	Strigidae (Owls)
183	*Otus bakkamoena*	Strigiformes	Strigidae (Owls)
184	*Otus sunia*	Strigiformes	Strigidae (Owls)
185	*Strix leptogrammica*	Strigiformes	Strigidae (Owls)
186	*Strix ocellata*	Strigiformes	Strigidae (Owls)
187	*Phodilus assimilis*	Strigiformes	Tytonidae (Barn-Owls)
188	*Harpactes fasciatus*	Trogoniformes	Trogonidae (Trogons)

Table S2 Top species contributing to each cluster at optimal value of K=5 for Ecostructure analyses

**Cluster1**	**Cluster2**	**Cluster3**	**Cluster4**	**Cluster5**
Otus bakkamoena	*Oriolus kundoo*	*Cinnyris lotenius*	*Dendrocopos macei*	*Gracula indica*
Parus cinereus	*Zoonavena sylvatica*	*Aethopyga vigorsii*	*Anthracoceros albirostris*	*Lyncornis macrotis*
Centropus sinensis	*Salpornis spilonota*	*Pycnonotus xantholaemus*	*Caprimulgus macrurus*	*Ducula cuprea*
Argya striata	*Pitta brachyura*	*Ninox scutulata*	*Rhipidura albicollis*	*Sturnia blythii*
Spilopelia chinensis	*Sitta castanea*	*Turdus simillimus*	*Aethopyga siparaja*	*Rubigula gularis*

Table S3 Model rankings for Spatial Autoregressive models based on AIC and ∆AIC scores

Response variable	Model	AIC	∆AIC
Species Richness (SR)	Climate+Topography	23003	0
	Climate	23209.19	206.19
	Topography	24325.79	1322.79
Phylogenetic Diversity (PD)	Climate+Topography	-9533.47	0
	Climate	-8980.47	553
	Topography	-7890.65	1642.82

Phylogenetic Endemism (PE)	Climate+Topography	-46432.4	0
	Climate	-46194.7	237.7
	Topography	-44658.9	1773.5

Table S4 Species with no phylogenetic information in the McTavish et al. 2025 global bird phylogeny

**Sno**	**Species**
1	*Sturnia blythii*
2	*Rhipidura albogularis*
3	*Chrysocolaptes socialis*
4	*Tephrodornis sylvicola*
5	*Columba elphinstonii*
6	*Columba punicea*
7	*Montecincla meridionalis*
8	*Montecincla jerdoni*
9	*Ketupa coromanda*
10	*Leptocoma zeylonica*
11	*Leptocoma minima*

12	*Aethopyga vigorsii*
13	*Treron affinis*
14	*Dendrocitta leucogastra*
15	*Machlolophus aplonotus*
16	*Chrysocolaptes festivus*
17	*Nisaetus kelaarti*
18	*Strix ocellata*
19	*Sitta castanea*
20	*Myophonus horsfieldii*
21	*Caprimulgus atripennis*
22	*Yungipicus nanus*
23	*Athene blewitti*
24	*Cinnyris lotenius*
25	*Ducula cuprea*
26	*Aviceda jerdoni*
27	*Zoonavena sylvatica*
28	*Hirundapus giganteus*
29	*Phodilus assimilis*

References

McTavish, Emily Jane, Jeff A. Gerbracht, Mark T. Holder, Marshall J. Iliff, Denis Lepage, Pamela C. Rasmussen, Benjamin D. Redelings, Luna L. Sánchez Reyes, and Eliot T. Miller. 2025. "A Complete and Dynamic Tree of Birds." Proceedings of the National Academy of Sciences of the United States of America 122 (18): e2409658122. https://doi.org/10.1073/pnas.2409658122.

Nitta, Joel H., Shawn W. Laffan, Brent D. Mishler, and Wataru Iwasaki. 2023. "Canaper: Categorical Analysis of Neo- and Paleo-endemism in R." *Ecography* 2023 (9). https://doi.org/10.1111/ecog.06638.

Tobias, Joseph A., Catherine Sheard, Alex L. Pigot, Adam J. M. Devenish, Jingyi Yang, Ferran Sayol, Montague H. C. Neate-Clegg, et al. 2022. "AVONET: Morphological, Ecological and Geographical Data for All Birds." *Ecology Letters* 25 (3): 581–97.
